# The NAC transcription factor FaRIF controls fruit ripening in strawberry

**DOI:** 10.1093/plcell/koab070

**Published:** 2021-02-24

**Authors:** Carmen Martín-Pizarro, José G Vallarino, Sonia Osorio, Victoriano Meco, María Urrutia, Jeremy Pillet, Ana Casañal, Catharina Merchante, Iraida Amaya, Lothar Willmitzer, Alisdair R Fernie, James J Giovannoni, Miguel A Botella, Victoriano Valpuesta, David Posé

**Affiliations:** 1 Laboratorio de Bioquímica y Biotecnología Vegetal, Instituto de Hortofruticultura Subtropical y Mediterránea (IHSM), Universidad de Málaga-Consejo Superior de Investigaciones Científicas, Departamento de Biología Molecular y Bioquímica, Facultad de Ciencias, UMA, Málaga, Spain; 2 Unidad Asociada de I+D+i IFAPA-CSIC Biotecnología y Mejora en Fresa, Málaga, Spain; 3 Laboratorio de Genómica y Biotecnología, Centro IFAPA de Málaga, Instituto Andaluz de Investigación y Formación Agraria y Pesquera, 29140 Málaga, Spain; 4 Max-Planck-Institute of Molecular Plant Physiology, Potsdam-Golm 144776, Germany; 5 United States Department of Agriculture and Boyce Thompson Institute for Plant Research, Cornell University, Ithaca, NY 14853, USA

## Abstract

In contrast to climacteric fruits such as tomato, the knowledge on key regulatory genes controlling the ripening of strawberry, a nonclimacteric fruit, is still limited. NAC transcription factors (TFs) mediate different developmental processes in plants. Here, we identified and characterized Ripening Inducing Factor (FaRIF), a NAC TF that is highly expressed and induced in strawberry receptacles during ripening. Functional analyses based on stable transgenic lines aimed at silencing *FaRIF* by RNA interference, either from a constitutive promoter or the ripe receptacle-specific *EXP2* promoter, as well as overexpression lines showed that FaRIF controls critical ripening-related processes such as fruit softening and pigment and sugar accumulation. Physiological, metabolome, and transcriptome analyses of receptacles of *FaRIF-*silenced and overexpression lines point to FaRIF as a key regulator of strawberry fruit ripening from early developmental stages, controlling abscisic acid biosynthesis and signaling, cell-wall degradation, and modification, the phenylpropanoid pathway, volatiles production, and the balance of the aerobic/anaerobic metabolism. FaRIF is therefore a target to be modified/edited to control the quality of strawberry fruits.

## Teaser

We identified and characterized the NAC transcription factor FaRIF, which plays an essential role in the regulation of different processes associated with strawberry fruit ripening.

## INTRODUCTION

Strawberry (*Fragaria ×* *ananassa* Duch.) is one of the most popular fruit crops thanks to the unique flavor and aroma of its berries, two critical quality parameters that are acquired during the ripening process. The strawberry fruit is an achenetum, consisting of a fleshy part (receptacle) that results from the development of the flower receptacle, in which the actual fruits (achenes) are embedded (Liu et al., 2020). Strawberry ripening is a genetically programmed and highly coordinated process that leads to structural and biochemical changes such as receptacle softening and increase in the contents of sugars, anthocyanins, volatile compounds, and vitamins. Strawberry fruit has been considered as a genuine example of nonclimacteric fruit ripening, which, in contrast to that of climacteric fruits, does not require ethylene to initiate and/or maintain the ripening program (Symons et al., 2012). Nevertheless, several reports have found that the application of ethylene to strawberry fruits and the generation of plants with reduced ethylene sensitivity have an effect on strawberry ripening (Trainotti et al., 2005; Villareal et al., 2010; Sun et al., 2013; Merchante et al., 2013). The main phytohormones controlling the enlargement of the strawberry fruit receptacle at early stages are auxin and gibberellic acid (GA) (Nitsch, 1950; Csukasi et al., 2011; Kang et al., 2013; Estrada-Johnson et al., 2017; Liao et al., 2018), whereas abscisic acid (ABA) is considered the main phytohormone controlling the ripening process (Jia et al., 2011). Besides ABA, other phytohormones have been proposed to be involved in specific molecular processes associated with ripening in receptacles and/or achenes such as ethylene (Merchante et al., 2013), jasmonate (Concha et al., 2013), brassinosteroids (Chai et al., 2012), and polyamines (Guo et al., 2018). 

**Figure:**
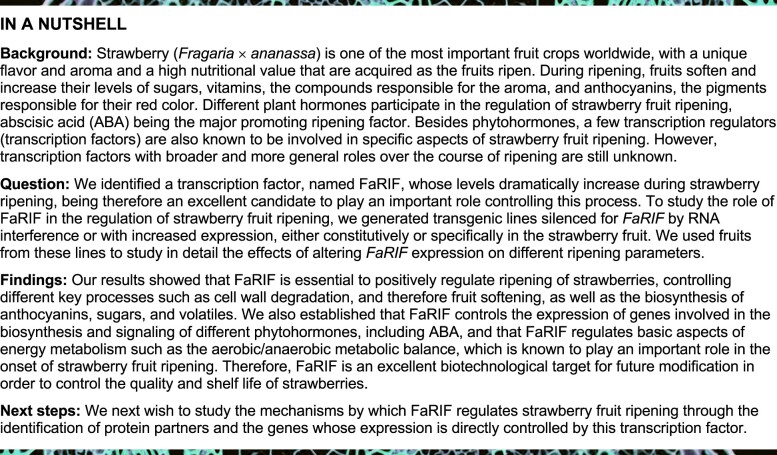


Besides the role of different phytohormones in the control of ripening, a number of TFs have also been identified as important regulators. Among them, several TFs belonging to the MYB family have been identified and shown to be involved in regulating flavonoid accumulation such as the positive regulator MYB10 (Lin-Wang et al., 2010; Medina-Puche et al., 2013; Castillejo et al., 2020), and the repressor MYB1 (Aharoni et al., 2001), and in the biosynthesis of flavonoids and ABA (GA-induced MYB; GAMYB) (Vallarino et al., 2015), sucrose (GAMYB and MYB44.2) (Wei et al., 2018), and the volatile compound eugenol (EMISSION OF BENZENOIDS II; EOBII, which acts together with the DOF-like TF FaDOF2) (Medina-Puche et al., 2015; Molina-Hidalgo et al., 2017). In addition, other TFs have been proposed to play a role in fruit setting and ripening, such as the basic Helix–Loop–Helix (bHLH) SPATULA (FaSPT) (Tisza et al., 2010), the atypical HLH PACLOBUTRAZOL RESISTANCE1 (FaPRE1) (Medina-Puche et al., 2019), the MADS-box SHATTERPROOF-like (FaSHP) (Daminato et al., 2013), and the SEPALLATA1/2-like (FaMADS9) (Seymour et al., 2011; Vallarino et al., 2019). However, a general regulator of strawberry ripening process has not been described so far.

NAC (NAM, ATAF, and CUC) TFs constitute a large protein family that plays important regulatory roles in plant development and environmental responses (Olsen et al., 2005). These TFs are characterized by a conserved region known as the NAC domain, located at their N terminus and involved in DNA recognition, dimerization, and binding, whereas their C terminus is highly diverse and determines the different NAC subgroups (Ooka et al., 2003). Members of this family are involved in the regulation of ripening-associated processes in fruits such as citrus (de Oliveira et al., 2011), banana (*Musa acuminata*) (Shan et al., 2012), tomato (*Solanum lycopersicum*) (Zhu et al., 2014; Kou et al., 2016), peach (*Prunus persica*) (Zhou et al., 2015), kiwifruit (*Actinidia deliciosa*) (Nieuwenhuizen et al., 2015), and apple (*Malus domestica*) (Yeats et al., 2019; Zhang et al., 2020). Recently, a total of 112 *NAC* genes were described in *F. vesca*, six of them with a potential role in the ripening process (Moyano et al., 2018). A genome-wide expression analysis of wild strawberry (*F. vesca*) *NAC* genes during abiotic and biotic stress has also been recently performed (Zhang et al., 2018). Moreover, the response to phytohormone treatments of some *NAC*s has also been tested (Moyano et al., 2018; Carrasco-Orellana et al., 2018). Among them, the expression of *FcNAC1* and the ortholog of *F. vesca* and *F. ×* *ananassa* *NAC022* in beach strawberry (*F. chiloensis*) responded to ABA and auxin. FcNAC1 also activated the expression of a cell-wall remodeling enzyme, pectate lyase (*FcPL*) in vitro (Carrasco-Orellana et al., 2018)*.* Despite all these data, there is no functional study for any NAC TF involved in strawberry fruit ripening to date.

In this study, we functionally characterized *FaNAC035*, which we renamed *FaRIF* (Ripening Inducing Factor). *FaRIF* encodes an NAC TF with the highest expression throughout strawberry fruit ripening (Sánchez-Sevilla et al., 2017). Stable transgenic lines overexpressing and silencing *FaRIF* under a constitutive promoter (cauliflower Mosaic Virus 35S) and stable lines silencing *FaRIF* from a fruit receptacle-specific promoter (*EXPANSIN2*) were established and phenotypically characterized, resulting in a clear alteration of different ripening-related parameters. Comprehensive metabolome and transcriptome analyses of the receptacle of *35S_pro_:RIF-*RNAi fruits point to FaRIF as a central regulator of strawberry fruit ripening, controlling main ripening processes, such as the phenylpropanoid pathway, cell-wall structure, phytohormone metabolism, and the aerobic/anaerobic balance of the central carbon metabolism.

## RESULTS

### Identification of NAC TFs potentially regulating strawberry fruit ripening

**Figure 1:**
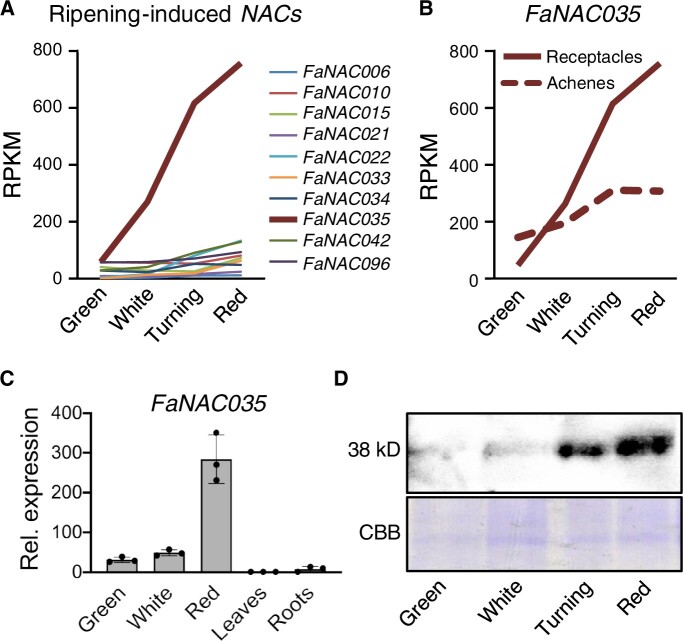
*FaNAC035* gene expression and protein levels dramatically increase during strawberry receptacle ripening. A, B, Expression pattern of ripening-induced NAC transcription factor genes at four ripening stages in receptacles (A), and of *FaNAC035* in receptacles and achenes (B); data from Sánchez-Sevilla et al. (2017). C, Relative expression of *FaNAC035* in wild-type fruit receptacles at three ripening stages, leaves and roots, as determined by RT-qPCR. Data are means ± se of three biological replicates. D, Immunoblot analysis in wild-type fruit receptacles at four ripening stages to detect FaRIF protein (38 kD) using anti-FaRIF antibody (upper). CBB staining of total nuclear protein extracts are shown in the bottom panel as loading control.

To identify candidate NAC TFs playing a role in the regulation of *F. ×* *ananassa* fruit ripening, we analyzed the expression of the 112 genes annotated as encoding NAC TFs in *F. vesca* (Moyano et al., 2018) using available transcriptome data from receptacles and achenes at four ripening stages (green, white, turning, and red), leaves and roots from *F.* × *ananassa* cv. Camarosa (Sánchez-Sevilla et al., 2017). Ten *NAC* genes showed increasing expression during ripening: *FaNAC006*, *FaNAC010, FaNAC015, FaNAC021*, *FaNAC022*, *FaNAC033*, *FaNAC034, FaNAC035*, *FaNAC042*, and *FaNAC096*, suggesting a putative role of their encoded TFs in the regulation of this process (Figure 1). Strikingly, *FaNAC035* was by far the *NAC* gene with the highest transcript levels among all ripening-induced *NAC* genes in *F.* × *ananassa* fruits (Figure 1A). Although the expression of *FaNAC035* in achenes was already rather high, it was even higher in receptacles (Figure 1B). We validated the expression pattern of *FaNAC035* by real-time quantitative PCR (RT-qPCR) analysis at three stages of ripening of receptacles, i.e., green, white, and red, and in two vegetative tissues, leaves, and roots (Figure 1C). Next, we generated specific antibodies against FaNAC035 and analyzed the amount of FaNAC035 protein by immunoblot analysis in receptacles during ripening (Figure 1D). Our results showed that FaNAC035 is present in receptacles at the green stage and that its level greatly increases during ripening (Figure 1D).

We next performed a phylogenetic analysis of FaNAC035 using the 112 NAC proteins from *F. vesca* (Moyano et al., 2018) as well as additional NACs belonging to different subgroups based on their C-terminal domain (Supplemental Figure S1 and Supplemental Data Set S1). Interestingly, FaNAC035 formed a monophyletic group with NACs involved in senescence, such as the NAM-B1 from peach (Guo and Gan, 2006) and the Arabidopsis NAC-REGULATED SEED MORPHOLOGY1 (NARS1) and NARS2 (Kunieda et al., 2008). FaNAC035 was also related to tomato NON-RIPENING (SlNOR), a TF-regulating climacteric ripening (Giovannoni, 2004; Wang et al., 2019), although the closest homolog to this tomato TF was FvNAC021.

Together, these data suggested that *FaNAC035* might play an important role in the regulation of strawberry fruit ripening. Thus, we selected *FaNAC035* for further characterization and named it as *FaRIF* (Ripening Inducing Factor).

### FaRIF promotes strawberry fruit ripening

**Figure 2:**
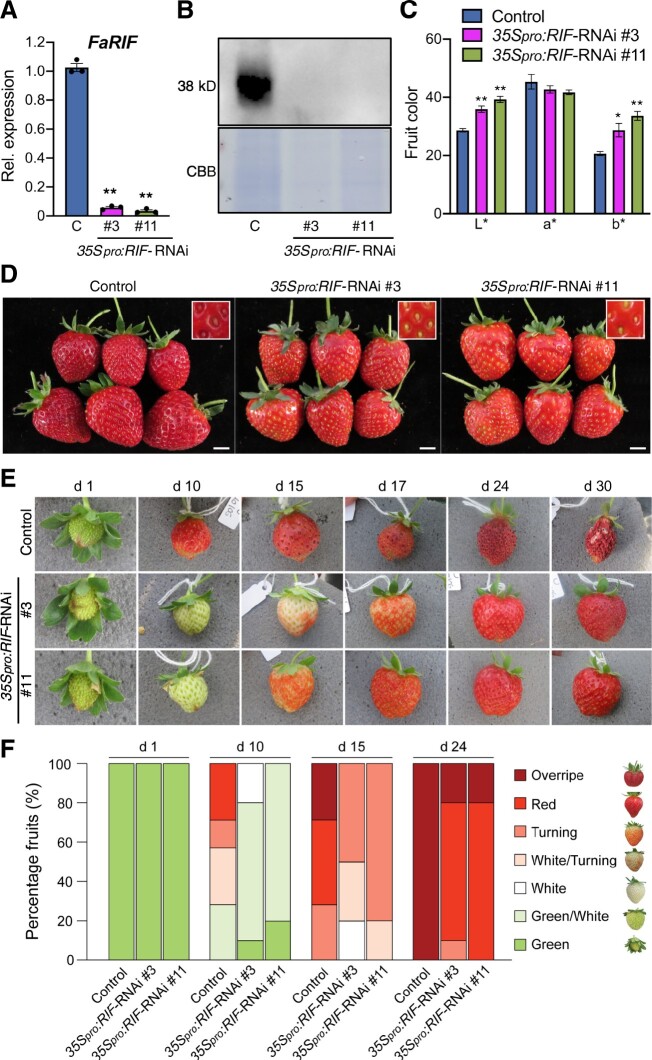
*35Spro:RIF-*RNAi lines show a strong silencing of *FaRIF* and display a paler red color and a delay in ripening progression. A, Relative expression of *FaRIF* in ripe receptacles from control (C) and stable *35Spro:RIF-*RNAi plants, as determined by RT-qPCR. B, Immunoblot analysis in ripe receptacles of control and stable *35Spro:RIF-*RNAi plants to detect FaRIF protein (38 kD) using anti-FaRIF antibody (upper). CBB staining of total nuclear protein extracts are shown in the bottom panel as loading control. C, Color characterization in the CIELAB color space for the lightness-darkness coefficient (L*), green-red (a*), and yellow-blue spectrum (b*). D, Fruit phenotype at the red stage in control and stable *35Spro:RIF-*RNAi transgenic lines. Inset: detail of the achenes. Scale bars, 1 cm. E, Representative pictures of a single fruit for each line at different times. A total of 10 fruits were marked at the same early green stage (Day 1), and the phenotypes were monitored 10, 15, 17, 24, and 30 days afterward. F, Percentage of fruits at each developmental/ripening stage for each time point. Ten fruits were analyzed per genotype. Data in (A) and (C) are means ± se of 3 and 10 biological replicates, respectively, analyzed by Student’s *t*-test (*P < 0.01; **P < 0.001).

We cloned the *FaRIF* cDNA from ripe *F.* × *ananassa* cv. Camarosa fruits. The predicted FaRIF protein differed in 22 out of 343 amino acids when compared with its *F. vesca* ortholog (*FvNAC035*; FvH4_3g20700) (Supplemental Data S1 and Figure S2). In order to investigate the role of *FaRIF*, we selected a specific 265-bp sequence downstream of the conserved NAC domain coding sequence and generated a construct for gene silencing by RNA interference (RNAi) (Supplemental Data S1 and Figure S2) driven by the constitutive 35S CaMV promoter (*35Spro:RIF-*RNAi). We transferred four independent lines with similar fruit phenotypes to the greenhouse (Supplemental Figure S3). We selected two lines, *35Spro:RIF-*RNAi #3 and #11, as representative lines based on their low levels of *FaRIF* mRNA (Figure 2A). Further analysis of FaRIF protein levels in red receptacles by immunoblot analysis detected no protein accumulation in any of the *35S_pro_:RIF-*RNAi lines (Figure 2B), indicating that *FaRIF* RNAi-mediated silencing was highly efficient.

Constitutive silencing of *FaRIF* did not result in altered vegetative growth or development (Supplemental Figure S4). However, visual analysis of ripe receptacles of *35S_pro_:RIF-*RNAi lines showed a lighter red color compared with control fruits at the same developmental stage (Figure 2D). We quantified color differences using the CIELAB color space values: a* (green-red spectrum), b* (blue-yellow spectrum), and L* (brightness-darkness). We observed significant differences between control and transgenic lines for L* and b*, but not for a*, indicating that silencing of *FaRIF* generates paler fruits whose color profile is enriched in the yellow part of the spectrum (Figure 2C). In addition to the receptacles, ripe achenes from the *35S_pro_:RIF-*RNAi lines were also lighter in color than those in the control, indicating a role of *FaRIF* in achene ripening.

Next, we compared fruits of *35S_pro_:RIF-*RNAi and control lines from green with overripe stages. For this purpose, we monitored fruits marked at the early green stage over time (Figure 2E) and calculated the percentages of fruits at different developmental stages (Figure 2F). We noticed a delay in color in *35S_pro_:RIF-*RNAi fruits compared with the control after 10 days of development. Moreover, this delay in ripening progression in the RNAi lines was maintained after 30 days. At this stage, 100% of the control fruits were clearly wilted and senescent, with some showing symptoms of infection by powdery mildew. In contrast, most of the silenced fruits were hydrated and appeared healthy at the red stage (Figure 2E). Thus, the fruit phenotypes and the delayed ripening of the RNAi lines indicated an important role of FaRIF in promoting ripening of strawberry fruits.

### FaRIF controls cell-wall metabolism and fruit firmness

To identify genes associated with *FaRIF* function in fruit ripening, we performed a transcriptome deep sequencing (RNA-seq) analysis using receptacles from control and *35S_pro_:RIF-*RNAi #3 and #11 lines at the white stage and at their maximum stage of ripening (hereafter called red stage). We mapped RNA-seq reads to the v4.0.a1 assembly and annotation of the *F. vesca* reference genome (Edger et al., 2018). A principal component analysis showed that PC1 separated the samples based on the developmental stages, whereas PC2 clustered the samples according to their genotypes, as *35S_pro_:RIF-*RNAi #3 and #11 lines shared a similar transcriptome that was quite distinct from the control (Supplemental Figure S5A).

We calculated normalized read counts (reads per kilobase of transcript per million, RPKM) for each gene and removed genes with RPKM values lower than 1 in all the samples. With this threshold, 15,790 genes were deemed expressed from a total of 28,588 annotated genes (Supplemental Data Set S1). To determine differentially expressed genes (DEGs) between the control and both RNAi lines, we selected only genes with a false discovery rate *P*-value correction ≤0.05 for each line and stage separately (Supplemental Figure 5B). We retained 1,368 DEGs at the white stage and 1,535 DEGs at the red stage, of which 367 were differentially expressed in both lines at the two ripening stages (Supplemental Figure S5B, Data Set S2).

**Figure 3:**
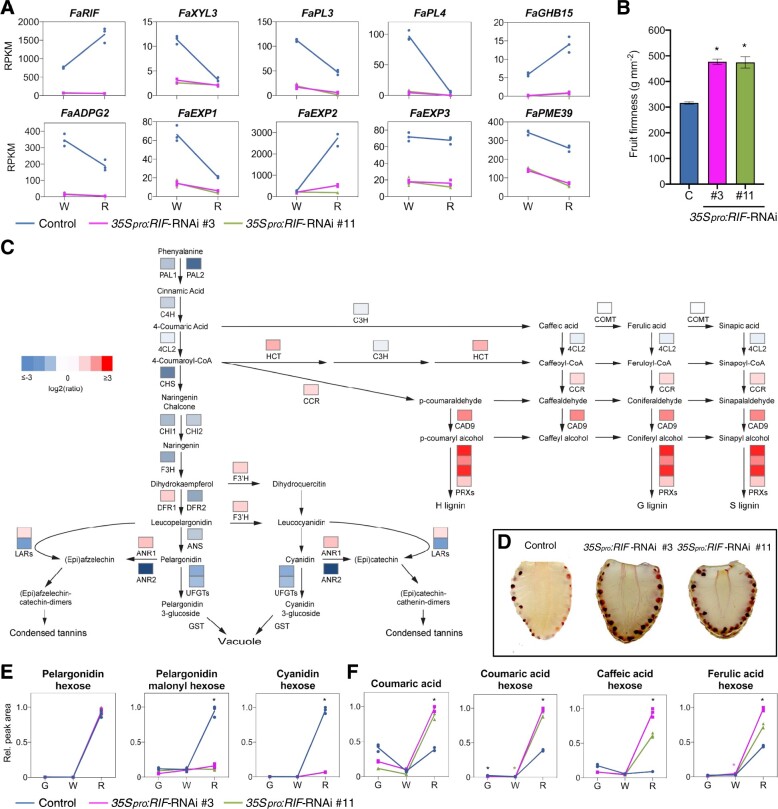
FaRIF controls the expression of genes involved in cell-wall degradation and the phenylpropanoid pathway, controlling fruit firmness, and anthocyanin and lignin levels. A, Expression of *FaRIF* and genes involved in cell-wall degradation and modification in control and *35Spro:RIF-*RNAi white (W) and red (R) receptacles. B, Fruit firmness measurement in control (C) and *35Spro:RIF-*RNAi ripe receptacles. Data are means ± se of 10 biological replicates analyzed by Student’s *t-*test (*P < 0.001). C, Phenylpropanoid, flavonoid, and lignin biosynthetic pathways. Colors denote the average of the log_2_ of the *35Spro:RIF-*RNAi/control expression ratio in both transgenic lines at the red stage for the respective genes. Red and blue show up- and downregulation, respectively, in both silenced lines. D, Lignin staining in ripe fruit sections from control and *35Spro:RIF-*RNAi plants using phloroglucinol. Photographs were taken at the same distance. E, F, Changes in relative contents of anthocyanins (E) and hydroxycinnamic acid derivatives (F) in green (G), white (W), and red (R) receptacles of control and *35Spro:RIF-*RNAi receptacles. Data in (E) and (F) were analyzed by Student’s *t* test (*P < 0.0005). Black asterisks indicate *P* < 0.0005 for both RNAi lines compared with the control. Colored asterisks denote *P* < 0.0005 for one of the RNAi lines compared with the control.

The transcriptome analysis confirmed that the transcript levels of *FaRIF* (*FaNAC035*) were dramatically reduced in both transgenic lines, i.e. approximately 10- and ∼12-fold reduction in lines #3 and #11, respectively, at the white stage, and ∼28- and ∼25-fold for lines #3 and #11, respectively, at the red stage (Figure 3A;Supplemental Data Set S3), indicating that the silencing mediated by the RNAi construct under the *35S* promoter is very efficient. In addition to *FaRIF*, 5 out of the 10 ripening-related *NAC* genes were differentially expressed in at least one ripening stage but only *FaNAC042* was strongly downregulated in both RNAi lines at the two ripening stages (Supplemental Data Set S3). To evaluate the possibility of a possible nonspecific silencing of *FaNAC042*, we analyzed the similarity between the *FaRIF* RNAi hairpin sequence and the three *FaNAC042* homeolog sequences in *F. × ananassa*. As shown in Supplemental Figure S6, the hairpin sequence is rather different than the sequences of *FaNAC042*, suggesting that *FaNAC042* expression is regulated either directly or indirectly by FaRIF instead of an unspecific silencing. Besides these *NAC* genes, other ripening-related TF genes, as the R2R3 MYB TF *FaEOBII*, the DOF-like *FaDOF2*, and two bHLHs, i.e. *FaSPT* and *FaPRE1*, were also downregulated in the *35S_pro_:RIF-*RNAi lines (Supplemental Data Set S1).

Next, we used MapMan bins (Usadel et al., 2009) in order to identify categories of genes or pathways enriched among the DEGs identified with a more than two-fold up- or downregulation in the RNAi lines (Supplemental Data Set S4). The decrease in strawberry fruit firmness during ripening is due to the activity of cell-wall degradation and remodeling enzymes (Posé et al., 2011). Fittingly, at the white stage, “cell wall” was one of the most enriched MapMan category, with several subcategories significantly enriched as well (Supplemental Figure S7 and Data Set S4). Thirteen out of the 16 genes of the “cell-wall degradation” subcategory were downregulated in *35S_pro_:RIF-*RNAi white receptacles, including genes encoding for a β-xylosidase (*FaXYL3*), two pectate lyases (*FaPL3* and *FaPL4*), an endo-1,4-beta-glucanase (*FaGH9B15*), and a polygalacturonase (*ARABIDOPSIS DEHISCENCE ZONE POLYGALACTURONASE* 2; *FaADPG2*), three of which (*FaPL3*, *FaGH9B15*, and *FaADPG2*) being also downregulated at the red stage (Figure 3A). Genes related to cell-wall modification were also downregulated in the RNAi lines, such as expansins (*FaEXP1*, *FaEXP2*, and *FaEXP3*), a pectin methylesterase (*FaPME39*), and arabino galactan-proteins (*FaAGP*s) (Dotto et al., 2006; Xue et al., 2020; Pérez-Pérez et al., 2018) (Figure 3A). Interestingly, other genes associated with cell-wall disassembly were upregulated in the RNAi lines such as pectate lyase 2 (*FaPL2/plB*), polygalacturonase 1 (*FaPG1*), *FaPME38*, and rhamnogalacturonate lyase 1 (*FaRGlyase1*) (Jiménez-Bermúdez et al., 2002; Benítez-Burraco et al., 2003; Quesada et al., 2009; Xue et al., 2020; Molina-Hidalgo et al., 2013) (Supplemental Figure S8A). These data support a significant modification of the cell-wall composition and assembly in *FaRIF*-RNAi fruits. Consistent with the altered expression of these cell-wall-related genes and the delayed ripening phenotype, the receptacles of the *35S_pro_:RIF-*RNAi lines were significantly firmer than those of the control (Figure 3B).

### FaRIF regulates the phenylpropanoid pathway, the accumulation of anthocyanins and lignin, and the expression of aroma-related genes

Changes in the content of phenolic compounds have been widely reported during strawberry fruit ripening, including anthocyanins, which are responsible for the red color of mature receptacles and achenes (Fait et al., 2008; Härtl et al., 2017). In agreement, MapMan analysis showed that the “secondary metabolism” bin was significantly enriched during the red stage, including numerous genes involved in the phenylpropanoid pathway (Supplemental Figure S7B and Data Set S4). Indeed, key genes in the initial steps of the pathway were significantly downregulated in red fruits of *35S_pro_:RIF-*RNAi plants, including the phenylalanine ammonia lyases genes *PAL1* and *PAL2*, cinnamic acid 4-hydroxylase (*C4H*), and 4-coumaroyl-CoA ligase (*4CL2*) (Figure 3C). Interestingly, the branching point leading to either the flavonoid or monolignol pathways showed a differential pattern. *CHS1*, encoding chalcone synthase 1, which is responsible for the first committed step in the flavonoid pathway, and most of the downstream genes were downregulated. Conversely, genes of the monolignol pathway, such as those encoding for hydroxycinnamoyltransferase, the cinnamoyl-CoA reductase, and the cinnamyl alcohol dehydrogenase 9, were upregulated (Figure 3C), suggesting an enhanced activity of this branch of the pathway.

In order to analyze to what extent these transcriptome changes might affect the profile of secondary metabolites, we performed a metabolome analysis using ultra performance liquid chromatography coupled to tandem mass spectrometry, extending the analysis to green, white, and red developmental stages (Supplemental Figure S9, Data Set S5). We detected significant changes in some anthocyanins and phenolic acids and their derivatives at the red stage (Figure 3, E and F; Supplemental Figure S9A). In particular, we observed a drastic reduction for cyanidin hexose, one of the major anthocyanidins responsible of the reddish-purple color and the minor pelargonidin malonyl hexose in the transgenic lines in comparison to the control (Figure 3E). However, we saw no differences for pelargonidin hexose or pelargonidin rutinose, responsible for the orange color. These changes may thus explain the lighter color and the enrichment in the yellow part of the color spectrum observed in ripe *FaRIF*-silenced fruits (Figure 2, C and D). Consistent with the transcriptome data, red receptacles of the RNAi lines showed an increase in the precursors of lignin biosynthesis, such as coumaric acid and the hexose derivatives of the coumaric, caffeic, and ferulic acids (Figure 3F). To determine whether the different hydroxycinnamic acid levels resulted in differences in lignin content, we performed histochemical staining of red fruits of control and *35S_pro_:RIF-*RNAi lines. As shown in Figure 3D, receptacles of the *35S_pro_:RIF-*RNAi lines showed increased staining relative to control lines in the vasculature and achenes, indicating a higher lignification in ripe *RIF*-silenced fruits.

Volatiles play an important role in the aroma of strawberry fruits. The accumulation of these compounds is closely related to changes in secondary metabolism during fruit ripening. To test whether FaRIF plays any role in the regulation of volatile production, we analyzed the expression in the receptacles of *35S_pro_:RIF-*RNAi lines of a number of genes reported to be involved in volatile biosynthesis in strawberry. Interestingly, the expression of *FaEOBII* and *FaDOF2*, which act synergistically to activate the transcription of *EUGENOL SYNTHASE2* (*FaEGS2*) and positively regulate the biosynthesis of the phenylpropanoid eugenol (Molina-Hidalgo et al., 2017), was downregulated in red fruits from the *35S_pro_:RIF-*RNAi lines (Supplemental Figure S8B). Furthermore, the expression of *FaEGS2* was also downregulated in ripe *FaRIF*-silenced fruits (Supplemental Figure S8B). Moreover, the red receptacles of *35S_pro_:RIF-*RNAi lines also showed a lower expression of *NEROLIDOL SYNTHASE1* (*FaNES1*) (Supplemental Figure S8B), involved in the biosynthesis of the terpenic volatile compounds linalool and nerolidol (Aharoni et al., 2004). These results suggest that FaRIF might be important to control the biosynthesis of compounds responsible for strawberry fruit aroma during the ripening process.

### FaRIF regulates phytohormone cross-talk to control ripening

**Figure 4:**
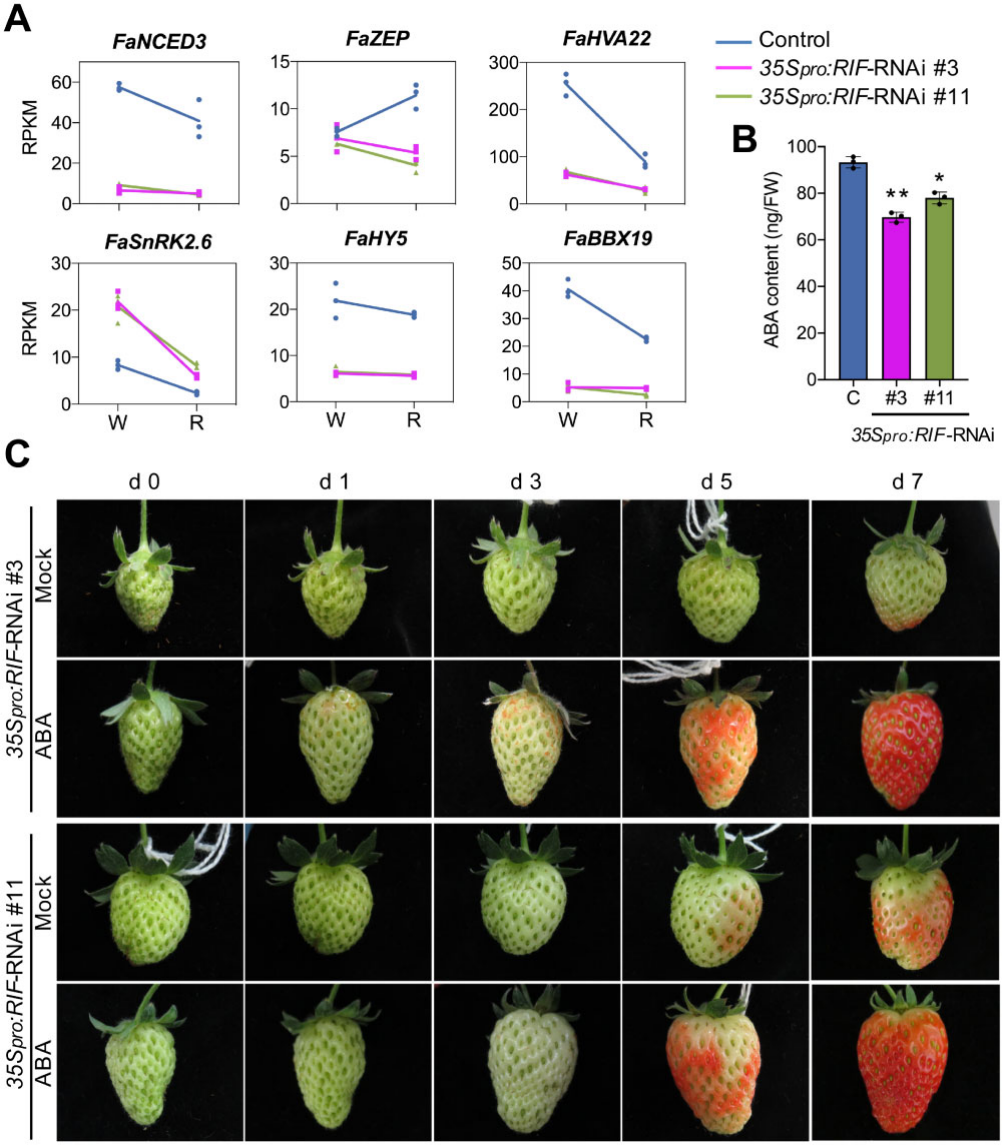
FaRIF regulates ABA biosynthesis and response. A, Expression of ABA biosynthetic (*FaNCED3* and *FaZEP*) and -responsive (*FaHVA22* and *FaSnRK2.6*) genes in control and *35Spro:RIF-*RNAi white (W) and red (R) receptacles. B, ABA content (ng/FW) in control (C) and *35Spro:RIF-*RNAi red receptacles. Data are means ± se of three biological replicates, analyzed by Student’s *t* test (*P < 0.005; **P < 0.0005). C, Ripening progression in *35Spro:RIF-*RNAi receptacles 7 days after mock-infiltration with 2% ethanol or 100 μM ABA. Representative pictures of a single fruit for each *35Spro:RIF-*RNAi line in each condition out of three biological replicates are shown. Fruit size cannot be directly compared across panels.

Strawberry fruit development and ripening are coordinated by a tightly controlled phytohormonal cross-talk (Liao et al., 2018; Gu et al., 2019). Our transcriptome analysis showed that the “Hormone metabolism” MapMan bin was also significantly enriched among DEGs between *35S_pro_:RIF-*RNAi and control receptacles at both ripening stages (Supplemental Figure S7 and Data Set S4). This category included genes related to the metabolism and signaling of ABA, the main phytohormone controlling strawberry fruit ripening as well as auxin, ethylene, and polyamines (Supplemental Table S1). Interestingly, the gene encoding the rate-limiting enzyme in ABA biosynthesis, 9-cis-epoxycarotenoid dioxygenase (*FaNCED3*), was significantly downregulated in *FaRIF-*silenced fruits at both ripening stages (Figure 4). Furthermore, the most expressed *NCED* in strawberry receptacles, *FaNCED5* (Liao et al., 2018), also named *FaNCED2* (Gu et al., 2019), was also downregulated in red receptacles of the silenced lines, although it was only significant in line #11 (Supplemental Data Set S3). Moreover, the ABA biosynthetic encoding gene encoding zeaxanthin epoxidase (*FaZEP*) was downregulated at the red stage (Figure 4A). In addition, a number of genes involved in ABA signaling were also differentially expressed, including *FaHVA22*, the protein kinase-encoding gene *SUCROSE NONFERMENTING1-RELATED PROTEIN KINASE 2.6* (*FaSnRK2.6*), and two TF-encoding genes, the light-responsive TFs *ELONGATED HYPOCOTYL5* (*FaHY5*) and the B-box-containing protein 19 (*FaBBX19*) (Shen et al., 2001; Han et al., 2015; Chen et al., 2008; Bai et al., 2019) (Figure 4A;Supplemental Table S1).

The down- or upregulation in the expression of all these genes would support a lower ABA content in *35S_pro_:RIF-*RNAi receptacles, which in turn might result in altered ripening progress in these lines. To investigate this possibility, we quantified the concentration of ABA in ripe receptacles, revealing a significant reduction in ABA content of 16%–25% in *35S_pro_:RIF-*RNAi red receptacles compared with that of control fruits (Figure 4B). This result supports our hypothesis that *FaRIF* acts upstream of ABA to regulate strawberry fruit ripening. Infiltration of ABA in strawberry receptacles induces ripening, as reflected by color formation (Moyano-Cañete et al., 2013; Li et al., 2016). We thus infiltrated green receptacles of *35S_pro_:RIF-*RNAi lines with 100 µM ABA and mock solutions. Interestingly, receptacles infiltrated with ABA developed color faster than mock-infiltrated fruits (Figure 4C), supporting a role for *FaRIF* in controlling ripening-associated changes such as the biosynthesis of anthocyanins through the regulation of ABA biosynthesis.

In addition to ABA, other phytohormones play an important role in strawberry fruit development and ripening (Liao et al., 2018; Gu et al., 2019). Our transcriptome analysis identified a number of genes involved in auxin biosynthesis or signaling that were differentially expressed in both white and red *35S_pro_:RIF*-RNAi receptacles, such as the cytochrome P450 gene *CYP79B*, the TF-encoding gene *AINTEGUMENTA-LIKE6* (*AIL6*), or the auxin repressor gene *Aux/IAA 9* (*IAA9*) (Sugawara et al., 2009; Krizek et al., 2016; Ulmasov et al., 1997) (Supplemental Table S1). Supporting an altered ABA and auxin content in the RNAi receptacles, the transcript levels of three ABA- and auxin-responsive TF genes, i.e., *FaSHP*, *ABA-STRESS-RIPENING* (*FaASR*), and *FaNAC022*, the ortholog to *FcNAC1* (Daminato et al., 2013; Jia et al., 2016; Carrasco-Orellana et al., 2018), were also altered at both white and red stages (Supplemental Table S1). Furthermore, genes involved in the ethylene signaling pathway were upregulated, whereas genes involved in polyamines biosynthesis were downregulated in *35S_pro_:RIF*-RNAi white and red receptacles (Supplemental Table S1). These data support an important role for FaRIF in controlling the phytohormonal balance during strawberry fruit ripening not only by inducing ABA biosynthesis but also potentially by regulating the biosynthesis and signaling of other strawberry development and ripening-related phytohormones such as auxin, ethylene, and polyamines.

### FaRIF regulates primary metabolism inhibiting glycolysis and fermentation

**Figure 5:**
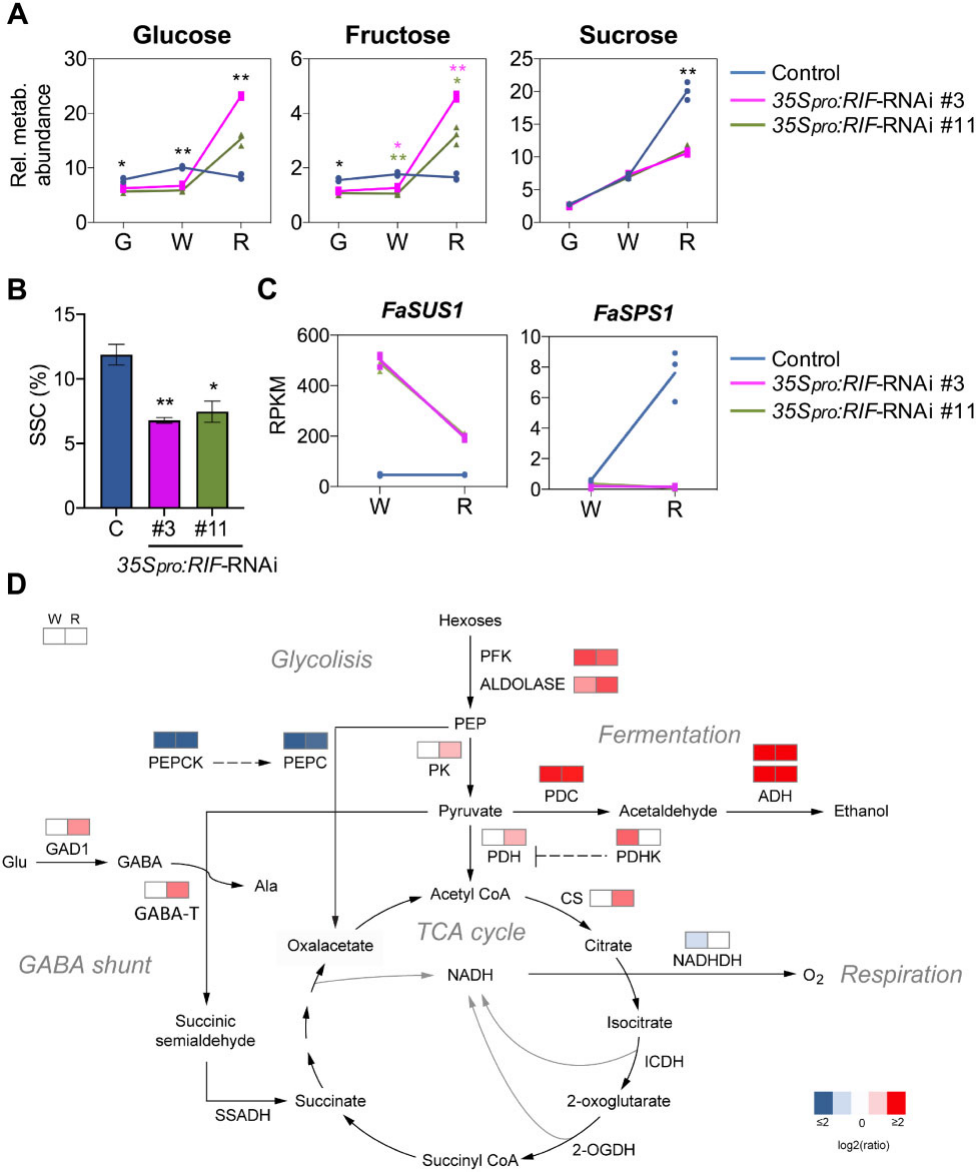
FaRIF contributes to the regulation of primary and energy metabolism. A, Content of main sugars in control and *35Spro:RIF-*RNAi receptacles at three ripening stages. Data are normalized to the mean response calculated for an internal control. B, Quantification of SSC. C, Expression of genes involved in sucrose metabolism supporting higher levels of glucose and fructose and lower levels of sucrose. D, Expression of genes involved in glycolysis, TCA cycle, fermentation, respiration, and GABA shunt by RNA-seq. Colors denote the average log_2_ fold-change of *35Spro:RIF-*RNAi/control in both transgenic lines, at the white and red stages. Red and blue show up- and downregulation, respectively, in both silenced lines. Data in (A) and (B) are from three and 10 biological replicates, respectively, analyzed by Student’s *t* test (*P < 0.01; **P < 0.001). Colored asterisks denote significant difference for one of the RNAi lines compared with the control. G, green; W, white; R, red receptacles.

Carbohydrate metabolism is another key process controlling fruit growth and development (Kanayama, 2017). A good example is sucrose, which, in addition to being a major determinant of fruit quality, functions as a signal promoting ABA accumulation and strawberry fruit ripening (Jia et al., 2013). Therefore, we analyzed primary metabolites in receptacles of control and *35S_pro_:RIF-*RNAi transgenic lines at the green, white, and red developmental stages by gas chromatography-mass spectrometry (GC–MS). Most of the metabolites analyzed showed differences in silenced fruits from both transgenic lines compared with the control, at least at one stage (Supplemental Figure S9B and Data Set S6). Remarkably, main sugar levels were altered in the RNAi lines. Indeed, although glucose and fructose levels significantly increased in red *35S_pro_:RIF-*RNAi receptacles, their sucrose content was significantly reduced (Figure 5A). These changes in main sugars were reflected in a lower content of soluble solids (soluble solid content, SSC or °Brix) in red *35Spro:RIF-*RNAi receptacles (Figure 5B). Consistent with this observation, *SUCROSE SYNTHASE1* (*FaSUS1*), which encodes the enzyme catalyzing the reversible cleavage of sucrose, was upregulated in both white and red receptacles of *35Spro:RIF-*RNAi fruits (Figure 5C). In addition, *SUCROSE PHOSPHATE SYNTHASE1* (*FaSPS1*), involved in sucrose biosynthesis, was dramatically downregulated (over 50-fold) at the red stage (Figure 5C).

Directly related to glucose and fructose metabolism, we determined that genes encoding glycolytic enzymes, such as *ATP-DEPENDENT 6-PHOSPHOFRUCTOKINASE6* (*FaPFK6*), *FRUCTOSE-BISPHOSPHATE ALDOLASE8* (*FaFBA8*), and *PYRUVATE KINASE* (*FaPK*), were upregulated in silenced fruits (Figure 5D). This result is consistent with an induced glycolytic flux, which is supported by a two-fold increase in the content of pyruvate (Supplemental Data Set S6). Pyruvate can follow different fates depending on the cellular environment, specifically energy demand and oxygen availability (Bui et al., 2019). Notably, MapMan analysis showed enrichment in genes involved in the fermentation process (Supplemental Figure S7B), such as *PYRUVATE DECARBOXYLASE1* (*FaPDC1*) and two *ALCOHOL* DEHYDROGENASE genes (*FaADHs*), which were upregulated in *35Spro:RIF-*RNAi receptacles (Figure 5D; Supplemental Table S1). Supporting an increase in anaerobic metabolism, two *ETHYLENE-RESPONSIVE FACTOR* (*ERF*) genes, *FaERF-74* and *FaERF-17* (Li et al., 2020), whose putative orthologs in Arabidopsis, *RELATED TO APETALA2 12* (*RAP2.12*) and *HYPOXIA-RESPONSIVE ERF* (*HRE2*), are positive regulators of the hypoxic response (Hinz et al., 2010; Licausi et al., 2010), were also upregulated in receptacles of RNAi lines (Supplemental Table S1). In parallel, genes involved in the tricarboxylic acid cycle were also differentially expressed, such as the genes encoding the mitochrondrion-localized enzymes PYRUVATE DEHYDROGENASE (FaPDH) and CITRATE SYNTHASE (FaCS), which were upregulated in red *35Spro:RIF-*RNAi, in agreement with the increase in the content of the tricarboxylic acid intermediates citric, 2-oxoglutaric, and malic acids (Figure 5D;Supplemental Table S1 and Figure S9B). Besides these changes, a number of genes involved in primary energetic metabolism were also differentially expressed, such as a *NADH DEHYDROGENASE* (*FaNADHDH*), *PYRUVATE DEHYDROGENASE KINASE* (*FaPDHK*), *PHOSPHOENOLPYRUVATE CARBOXYLASE* (*FaPEPC*), and *PHOSPHOENOLPYRUVATE CARBOXYLASE KINASE* (*FaPEPCK*), among other genes (Figure 5D;Supplemental Table S1), suggesting an alteration in the balance of aerobic/anaerobic metabolism in *35Spro:RIF*-RNAi fruits, a parameter that has been reported to change along with strawberry fruit ripening (Wang et al., 2017).

### Genetic manipulation of *FaRIF* expression reveals its essential role in fruit development and ripening

We showed that, although *FaRIF* mRNA levels were remarkably high in receptacles, they were also rather high in achenes, reaching over 300 RPKM at the white and red-ripening stages (Figure 1B). It has been well established that communication between achenes and receptacles is important to control strawberry fruit development and ripening (Nitsch, 1950; Liao et al., 2018). Therefore, we next aimed to study the phenotypic and molecular consequences of silencing *FaRIF* specifically in the receptacle during late stages of ripening. For this purpose, we placed the expression of the *FaRIF* RNAi hairpin under the control of the *EXPANSIN2* (*FaEXP2*) promoter (*EXP2pro:RIF-*RNAi). *EXP2*, which encodes a cell-wall hydrolytic enzyme, is mostly expressed in receptacles from the turning stage (Supplemental Figure S10) (Sánchez-Sevilla et al., 2017). In addition, we also investigated the role of *FaRIF* in fruit ripening by overexpressing the *FaRIF* coding sequence (CDS) under the constitutive 35S promoter. For this purpose, we cloned the *Green Fluorescent Protein* (*GFP*) gene downstream and in-frame of the *FaRIF* CDS to generate *35Spro:RIF-GFP*.

**Figure 6:**
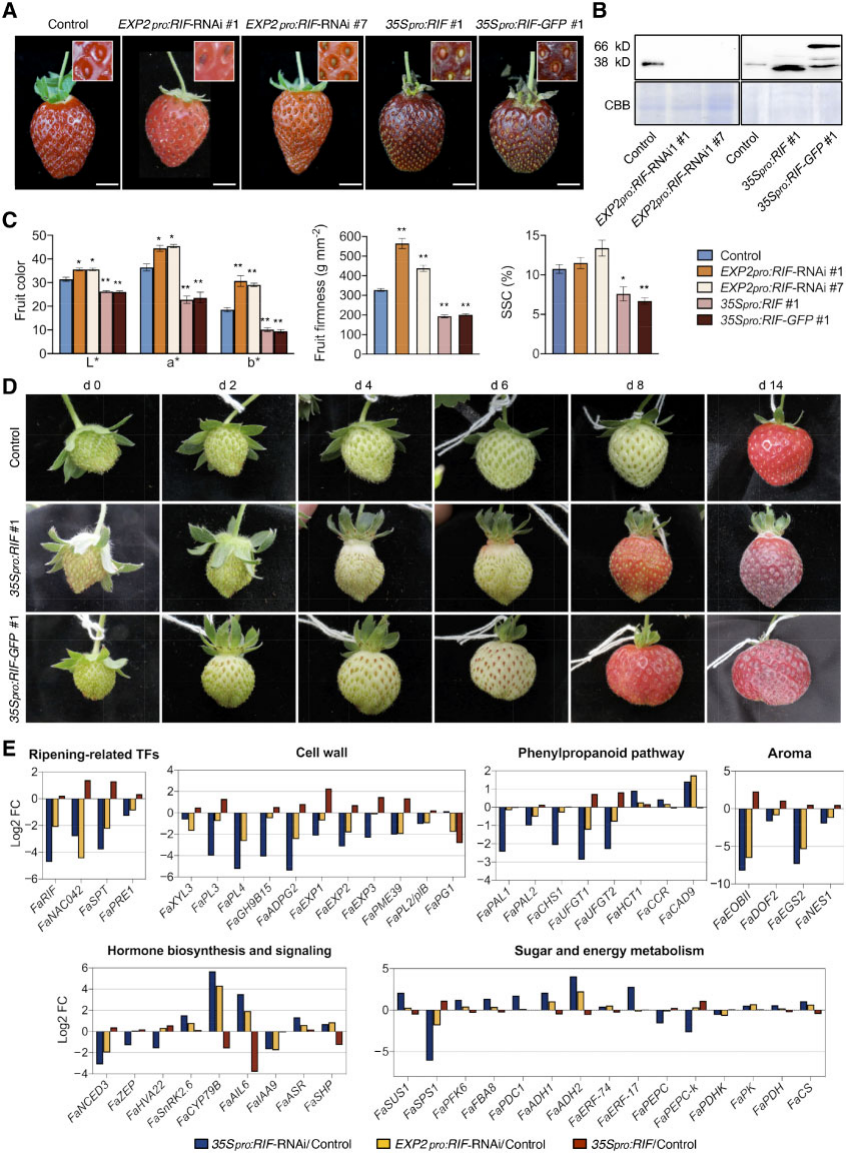
Effects on ripening when *FaRIF* is specifically silenced at late stages of receptacle ripening (*EXP2* promoter) or overexpressed (35S promoter). A, Fruit phenotype at the red stage in control and stable *EXP2pro:RIF-*RNAi, *35Spro:RIF* and *35Spro:RIF-GFP* transgenic lines. Inset: detail of the achenes. Scale bars, 1 cm. B, Immunoblot analysis in control and stable *EXP2pro:RIF-*RNAi, *35Spro:RIF* and *35Spro:RIF-GFP* ripe receptacles to detect native FaRIF protein (38 kD) and FaRIF–GFP fusion protein (66 kD) using anti-FaRIF antibody (upper). CBB staining of total nuclear protein extracts are shown in the (bottom) as loading control. C, Color characterization in the CIELAB color space for the lightness coefficient (L*), green-red (a*), and yellow-blue spectrum (b*) (left plot); fruit firmness measurements (middle plot); quantification of SSC (right plot). Data are means ± se of 10 biological replicates analyzed by Student’s *t* test (*P < 0.05; **P < 0.001). D, Representative pictures of a single fruit for control and *FaRIF* overexpression lines out of 10 analyzed showing the color progression over 14 days. Fruit size cannot be directly compared across panels. E, Log_2_ fold-change of genes involved in different processes in *35Spro:RIF-*RNAi, *EXP2pro:RIF-*RNAi, and *35Spro:RIF***.**

Two out of four established stable transgenic lines for *EXP2pro:RIF-*RNAi (#1 and #7) and the only two surviving overexpressing lines (*35Spro:RIF* #1 and *35Spro:RIF-GFP* #1) were selected for further characterization (Figure 6). We failed to detect FaRIF in red receptacles of any of the two *EXP2pro:RIF-*RNAi lines selected, as determined by Immunoblot analyses, indicating efficient silencing of *FaRIF* under this promoter at the ripe stage (Figure 6B). Conversely, the overexpression lines showed a significant increase of FaRIF in red receptacles, either as a native protein (*35Spro:RIF* #1) or as a GFP-tagged protein (*35Spro:RIF-GFP* #1) (Figure 6B). In contrast to *35Spro-* and *EXP2pro-*RNAi lines, adult plants overexpressing *FaRIF* exhibited impaired plant development (Supplemental Figure S4), suggesting that the ubiquitous expression of *FaRIF* has deleterious effects.

The phenotypic analysis of ripe receptacles showed that the two *EXP2pro-*RNAi lines showed paler receptacles enriched in the yellow part of the spectrum, similar to what we observed in the *35S-*RNAi lines (Figure 6A and C). Interestingly, *FaRIF* silencing from the *EXP2* promoter resulted in dark red achenes (Figure 6A) in contrast to *35Spro:RIF-*RNAi lines (Figure 2D), which is consistent with the differential silencing of *FaRIF* expected in *35Spro-* and *EXP2pro-*RNAi lines. On the contrary, *FaRIF* overexpression lines showed an opposite color phenotype for their receptacles compared with the RNAi lines (Figure 6A). The receptacles were unusually dark and enriched in the green and blue part of the spectrum (Figure 6C). Fruit firmness also followed an opposite trend when *FaRIF* was either silenced or overexpressed, since ripe *EXP2_pro_:RIF-*RNAi and *35Spro:RIF*(*-GFP*) receptacles were firmer and softer than those of the control, respectively (Figure 6C). These results confirm the role of *FaRIF* in controlling anthocyanin production and cell-wall characteristics during ripening. Surprisingly, and in contrast to *35Spro:RIF-*RNAi lines, we observed no changes for SSC in *EXP2pro-*RNAi receptacles, whereas the overexpression lines showed a lower content of soluble solids content (Figure 6C).

In order to determine whether the opposite fruit phenotype of the *FaRIF* overexpression lines is a consequence of an acceleration of the global process, we next scored the progress of fruit ripening of the overexpression lines over time, as performed previously for the *35Spro:RIF-*RNAi lines (Figure 2E). As shown in Figure 6D, overexpression of *FaRIF* was sufficient to cause a faster anthocyanin production and receptacle senescence, supporting a key role for FaRIF in controlling the fruit-ripening progress.

Finally, we performed a transcriptome analysis of receptacles of the control, *EXP2pro:RIF-RNAi* #1 and #7, and *35Spro:RIF* #1 lines at their respective ripe stages (Supplemental Data Set S1). Interestingly, many genes previously found to be differentially expressed in *35Spro:RIF-*RNAi receptacles were confirmed in the *EXP2pro-*RNAi lines, whereas displaying an opposite trend in the overexpression line. Among these genes, *FaNAC042*, and three other ripening-related TF genes, i.e., *FaEOBII*, *FaSPT*, and *FaPRE1*, were downregulated in both *35Spro-* and *EXP2pro-*RNAi lines and induced in *35Spro:RIF* #1 (Figure 6E). The opposite expression of *FaNAC042* in the RNAi and the overexpression line support the role of FaRIF in promoting *FaNAC042* expression. Consistent with the higher and lower firmness that the RNAi and overexpression receptacles displayed, respectively (Figures 3, B and 6, C), genes involved in cell-wall degradation and modification showed a remarkable opposite trend, being mainly downregulated with both RNAi strategies, but upregulated in the overexpression line (Figure 6E). This opposite pattern was also seen for genes in the phenylpropanoid pathway, as expected based on the different color of the receptacle shown by the RNAi and the overexpression lines (Figures 2, D and 6, A), and for genes involved in the production of volatiles, phytohormone biosynthesis and signaling, and sugar and energy metabolism (Figure 6E). Thus, all these data support the role of FaRIF as a key regulator of strawberry fruit ripening.

## DISCUSSION

A number of TFs that regulates different and specific aspects of strawberry fruit ripening have been characterized to date (Aharoni et al., 2001; Lin-Wang et al., 2010; Seymour et al., 2011). Out of the 112 *NAC* genes identified in *F. vesca* (Moyano et al., 2018), 10 showed increases in their mRNA levels during ripening (Moyano et al., 2018; Sánchez-Sevilla et al., 2017) (Figure 1A). Among them, only *FcNAC1*, the ortholog to *NAC022* in *F. chiloensis*, has been characterized in relation to strawberry ripening. The authors identified cis-regulatory elements able to respond to some phytohormones and reported that *FcNAC1* expression is regulated by ABA and auxin (Carrasco-Orellana et al., 2018). However, no functional analysis for any ripening-related NAC TF has been described in strawberry. In the present study, we functionally characterized *FaNAC035* (*FaRIF*). Phenotypic and molecular analyses of *FaRIF-*RNAi and *FaRIF*-overexpression lines showed that FaRIF is key to promote ripening of strawberry fruits through regulating phytohormone biosynthesis and signaling, ripening-related TFs, energy metabolism, and specific processes such as cell-wall remodeling, the phenylpropanoid pathway, and sugar content.

The involvement of different phytohormones on the strawberry fruit growth and ripening processes has been well established (Symons et al., 2012; Gu et al., 2019). The early stages of strawberry receptacle development are controlled by auxin and GAs that are synthesized in achenes (Nitsch, 1950; Csukasi et al., 2011). Therefore, in these early stages, achene-receptacle communication is essential. At later stages, the ripening of the receptacle is dependent on a decrease in auxin content and a local biosynthesis of ABA (Jia et al., 2011; Liao et al., 2018). Nevertheless, regulatory factors controlling these phytohormones remain elusive. Our analyses indicate that *FaRIF* regulates genes involved in phytohormone biosynthesis and signaling, suggesting a regulatory role in phytohormone cross-talk to promote strawberry fruit development and ripening. Indeed, our results showed that FaRIF promotes ABA biosynthesis by the induction of *FaNCED3* and *FaZEP*. The recovery of the ripening process in *35Spro:RIF-*RNAi fruits treated with an exogenous application of ABA shows that FaRIF acts upstream of ABA (Figure 4C). Conversely, it was reported that the transcript levels of *FaRIF* are substantially diminished after application of 1-nordihydroguaiaretic acid, an inhibitor of ABA biosynthesis (Moyano et al., 2018), revealing that *FaRIF* expression is positively regulated by ABA. Thus, we propose that *FaRIF* and ABA may act in a positive regulatory feedback loop to promote strawberry fruit ripening. This regulatory mechanism is documented in other systems such as seedling photomorphogenesis, whereby an NAC TF (ATAF2) and brassinosteroids are involved in a feedback regulation loop (Peng et al., 2015).

ABA controls anthocyanin biosynthesis in strawberry fruits (Jia et al., 2011, 2016). Interestingly, we observed differential progression in the color of receptacles and achenes of *RIF*-RNAi lines under either the 35S or the *EXP2* promoters (Figure 6A), indicating that FaRIF is also important to induce anthocyanin biosynthesis in achenes and that it controls the biosynthesis in receptacles independently of achene development.

One of the main processes associated with strawberry fruit ripening is the loss of firmness of receptacles due to the activity of cell-wall remodeling enzymes (Posé et al., 2011). Our transcriptome analyses indicate that FaRIF positively regulates many genes involved in cell-wall disassembly (Figures 3, A and 6, E), although other cell-wall-related genes were upregulated in the RNAi lines (Supplemental Figure S8A). Therefore, although we cannot predict the specific effects of *FaRIF* silencing on cell-wall structure or composition, the altered firmness of the fruits in both the *FaRIF*-RNAi and overexpression lines supports a general role for FaRIF in cell-wall degradation and the loss of firmness during ripening. Besides cell-wall composition, the degree of lignification is also associated with firmness in many fleshy fruits (Li et al., 2010). Our data showed that *FaRIF* represses genes from the monolignol pathway, consistent with the increased lignin accumulation observed in *35Spro:RIF-*RNAi fruits (Figures 3, C and 6, E). Therefore, *FaRIF* plays a key role in promoting fruit softening by regulating cell-wall remodeling enzymes and lignin content.

The regulation of central metabolic fluxes is essential during fruit development (Centeno et al., 2011; Colombié et al., 2015). In strawberry fruits, the expression of *NADHDH* genes peaks between the white and the red fruit stages during ripening (Sánchez-Sevilla et al., 2017), suggesting an increase in respiration at this transition. It has also been reported that the glycolytic pathway is inhibited during ripening and that arresting respiration through the silencing of subunit alpha of the pyruvate dehydrogenase gene *PDHE1α* accelerates the ripening process. These studies indicate that the anaerobic/aerobic balance of the energy metabolism is programmed along with strawberry fruit ripening (Wang et al., 2017; Luo et al., 2020). Our transcriptome data showed that *FaRIF* participates in the control of this balance, as reflected by the changes in mRNA levels of metabolic genes of anaerobic pathways such as glycolysis and fermentation, aerobic pathways like the TCA cycle and respiration as well as genes from the anaplerotic pathways PEPC/PEPCK and the γ-aminobutyric acid (GABA) shunt (Figures 5, D and 6, E). Furthermore, metabolic changes observed in the *35Spro:RIF-*RNAi lines, such as the accumulation of glucose, fructose, raffinose, galactinol, *myo*-inositol, proline, and TCA intermediates, are characteristics of a hypoxia-like response (Banti et al., 2013). All these data point to FaRIF as an important regulator of the onset of aerobic metabolism that occurs during strawberry fruit ripening.

There is a local sucrose metabolism in fruits, whereby the enzymes SPS and SUS, and the recently identified protein kinase FaSnRK1a, play essential roles (Miron and Schaffer, 2005). *35Spro:RIF-*RNAi receptacles showed lower content of sucrose and a reduced SSC. Transcriptome analysis showed that *FaRIF* promotes sucrose accumulation during ripening, likely by regulating the expression of *FaSPS1* and *FaSUS*. Surprisingly, SSC was also reduced in *RIF* overexpression lines, despite showing an opposite transcript accumulation pattern for these metabolic genes and a remarkable acceleration of ripening processes such as coloration and loss of firmness (Figure 6, C and D). This result might be explained by a slower progress in sucrose accumulation compared with that of anthocyanin and cell-wall degradation during the ripening process. Thus, the red *35Spro:RIF*(*-GFP*) receptacles might be delayed in terms of sucrose composition, despite their advanced color and firmness. In contrast, despite the similar ripening phenotype seen in *EXP2pro-*RNAi and *35Spro-*RNAi lines, we observed no differences in SSC in ripe *EXP2pro:RIF-*RNAi receptacles relative to controls (Figure 6C). Since *FaRIF* was silenced in the late stages of ripening in the *EXP2pro-*RNAi lines, we hypothesize that *FaRIF* is important to control sucrose accumulation during ripening from early stages of development.

The phylogenetic analysis of FaRIF protein showed that it is closely related to NACs involved in senescence. Among them, the redundant Arabidopsis *NARS1/NAC* and *NARS2/NAM* genes have been shown to regulate embryogenesis and, interestingly, silique senescence (Kunieda et al., 2008). Furthermore, the phylogenetic analysis also revealed the homology between FaRIF and SlNOR, a TF previously reported to control tomato fruit ripening (Giovannoni, 2004; Wang et al., 2019). Besides, it has been reported that MdNAC18.1, the ortholog to SlNOR in apple, regulates apple fruit ripening by controlling fruit firmness and harvest time (Migicovsky et al., 2016; Yeats et al., 2019). Thus, all these data support a role for these NAC TFs in the regulation of ripening not only in both dry and fleshy fruits but also in organs with different ontogenetic origins such as the tomato fruit and false fruits such as apple (pome) and strawberry (achenetum).

In summary, we show that the NAC TF FaRIF plays a central role in controlling strawberry fruit ripening. Its regulatory role is executed from the early stages of fruit development, contributing to the crosstalk among different phytohormones through fruit growth and ripening, and to basic processes of fruit development such as the balance between anaerobic/aerobic metabolism. In addition, the delayed ripening phenotype of *FaRIF*-silenced fruits, characterized by an increased fruit firmness and extended life, opens the door to investigating the phenotypic consequences of different levels of *FaRIF* expression, and their potential application in extending fruit shelf-life in strawberry breeding programs. Although the constitutive silencing of *FaRIF* reduced SSC in ripe fruits, a late modulation of its expression, such as that seen in the *EXP2pro-*RNAi lines, might result in improved fruit firmness without changes in the sugar content.

## Materials and methods

### Cloning of *FaRIF* and phylogenetic studies

The full-length CDS for FvH4_3g20700 in *F.* × *ananassa* (*FaNAC035*, named *FaRIF* in this work) was obtained from cDNA generated from ripe *F.* × *ananassa* cv. Camarosa fruits using P13 and P14 oligonucleotides (Supplemental Table S2).

Multiple sequence alignment of NAC proteins was performed using MUSCLE with the Seaview version 4 software (Gouy et al., 2010). The phylogenetic tree was inferred by the neighbor-joining method. A total of 1,000 bootstrap pseudo-replicates were used to estimate the reliability of internal nodes. Evolutionary distances were computed using the Poisson correction method. Tree inference was performed using MEGA version 7 (Kumar et al., 2016). The dataset comprised 112 previously reported NAC proteins obtained from Genbank (see Accession numbers section and Moyano et al., 2018).

DNA sequence alignment was performed using the Clustal Omega program (https://www.ebi.ac.uk/Tools/msa/clustalo/).

### Plasmids construction

All oligonucleotides used for plasmid construction are listed in Supplemental Table S2. All PCR reactions were performed using iProof™ high-fidelity DNA polymerase (Bio-Rad), and the constructs were verified by Sanger sequencing.

The intron-containing hairpin RNA (ihpRNA) *35Spro:RIF-*RNAi construct was generated using a 265-bp fragment of *FaRIF* (nts 678–942 from the ATG codon), amplified from cDNA prepared from red fruits using primers P649 and P650, which incorporate two restriction sites used for cloning into the pHANNIBAL vector. The ihpRNA fragment cloned in pHANNIBAL was then introduced into the pBINPLUS binary vector using SacI/NheI and SacI/XbaI restriction sites, respectively, obtaining the final pBINPLUS-*35Spro:RIF-*RNAi construct. The ihpRNA *EXP2pro:RIF-*RNAi construct was generated using the same 265-bp *FaRIF* fragment amplified from the *35Spro:RIF-*RNAi construct and the *EXP2pro:GUS-GFP* construct (pKGWFS7 backbone) (Schaart et al., 2011). To generate the *FaRIF* overexpression constructs pCM23 (*35Spro:RIF*) and pCM24 (*35Spro:RIF-GFP*), the full *FaRIF* CDS was amplified from cDNA prepared from red fruits using primers P8 and P13. The PCR products were first cloned into pCR8/GW/TOPO vectors (Life Technologies) to create pCM9 and pCM10, respectively, which were recombined through LR reaction (Gateway) into the pK7WG2D and pK7WG2 binary vectors, respectively, resulting in pCM23 and pCM24.

### Plant materials and stable transformation

*F.* × *ananassa* cv. Camarosa control and transgenic strawberry adult plants were grown and maintained in a shading house (IFAPA, Churriana, Málaga, Spain) and greenhouse under natural sunlight (IHSM, Málaga, Spain) conditions, using a mixture of universal substrate and river sand (3:1 [v/v]). In vitro plants were grown in phytotrons under cool-white light (at 15 µE) with a long-day photoperiod (16-h light/8-h dark) at 22°C.

For stable transformation of *F.* × *ananassa* cv. Camarosa, plants were micropropagated in N30K medium supplemented with 2.2 µM kinetin. Transformation was performed according to the protocol described by Barceló et al. (1998). Strawberry leaf discs were transformed with Agrobacterium (*Agrobacterium tumefaciens*) strain LBA4404 harboring the constructs described above. Regenerated shoots were selected on the same medium supplemented with 50 mg L^−1^ kanamycin and 500 mg L^−1^ carbenicillin.

### Fruit phenotypic analysis

*35Spro:RIF-*RNAi lines were evaluated during three consecutive growing seasons, and *EXP2pro-*RNAi and overexpression lines during two. Non-transformed cv. Camarosa plants were used as control. At least 10 fruits for each control and transgenic lines were harvested at the stage of full ripeness. The external color of the fruit was analyzed using the Color reader CR-10 PLUS (KONICA MINOLTA, Ramsey, NJ, USA). For fruit firmness, two measures on each side of the fruit were performed using a penetrometer (Effegi FDP500) with a 3-mm diameter cylinder needle. SSC or °Brix was measured with a digital refractometer (ATAGO PR32).

### Evaluation of fruit ripening progress

Ten fruits from each line (control, *35Spro:RIF-*RNAi, *35Spro:RIF*, and *35Spro:RIF-GFP*) were labeled at the same early green stage (Day 0), and the fruit phenotype was monitored every day for 30 days (*35Spro:RIF-*RNAi) or 14 days (overexpression lines). Percentage of *35Spro:RIF-*RNAi fruits at seven different ripening stages (green, green/white, white, white/turning, turning, red, and overripe) was calculated at d 1, 10, 15, and 24.

### Gene expression analysis by RT-qPCR

Total RNA was isolated as previously described (Gambino et al., 2008) from three biological replicates of receptacles after the removal of achenes for the control and transgenic lines. Each biological replicate consisted of three technical replicates of five receptacles. One microgram of total RNA was DNase I-treated (TURBO™ DNAse, Invitrogen) and first-strand cDNA was synthesized using oligo(dT) ant the iScript cDNA Synthesis kit (Bio-Rad). The resulting first-strand cDNA was diluted 25-fold and 4 µL was used as a template. Quantitative PCR was performed using SsoFast EvaGreen^®^ Supermix (Bio-Rad) and specific oligonucleotides (Supplemental Table S2) on a CFX96^TM^ Real-Time System (Bio-Rad). Relative expression values were calculated by ΔΔC_t_ method using *FaCHP1* as the reference gene for the RT-qPCR normalization (Clancy et al., 2013) (Supplemental Table S2).

### Protein extraction and immunoblot analysis

Receptacles after the removal of achenes of control fruits at the green, white, turning, and red stages, and from control and transgenic fruits at the state of full ripeness were used for this analysis. Nuclear protein extraction was performed as previously described (Bouyer et al., 2011). The proteins were separated on 10% sodium dodecyl sulphate–polyacrylamide gel electrophoresis SDS–PAGE and electroblotted using Tran-Blot Turbo Transfer System (Bio-Rad) onto polyvinylidene difluoride (PVDF) membranes (Immobilon-P, Millipore) following the manufacturer’s protocol. The membranes were blocked using 30 mL of blocking solution (5% fat-free powdered milk in Tris-buffered saline with 0.05% Tween-20 [TBST]) for 2 h. Then, the membranes were washed with TBST and incubated with the anti-RIF antibody (1:50 dilution) overnight. The anti-RIF antibody was designed using a FaRIF 15-amino acid peptide (amino acids 268–282 from Met: C-PNLYWNHDQEDEAGL-NH_2_) and generated by Eurogentec, S.A. Liége Science Park (Belgium). The secondary antibody used in this study was anti-Rabbit IgG whole molecule-HRP conjugate (1:14,000; A-0545, Sigma). Signal was detected using Clarity ECL Western Blotting Substrates (Bio-Rad) or SuperSignal West Femto Maximum Sensitivity Substrate (ThermoFisher). The images were acquired using the Chemidoc XRS+System (Bio-Rad). SDS–PAGE and immunoblotted PVDF membranes were stained with Coomassie Brilliant Blue (CBB) R 250 to confirm equal loading between the different samples.

### Transcriptome analysis by RNA-seq

RNA-seq and primary and secondary metabolome analyses were carried out using the same samples. Each biological replicate comprised a minimum of 20 receptacles. The RNA quality and concentration were validated and measured on a Bioanalyzer 2100 (Agilent Technologies Santa Clara, CA, USA), and the RNA integrity number values were >8.0 for all biological replicates. Strand specific mRNA libraries were generated for *35Spro:RIF-RNAi* and their respective control samples as described by Zhong et al. (2011). Paired-end Illumina mRNA libraries were generated for *EXP2pro:RIF-RNAi*, *35Spro:RIF*, and their respective control samples using the TruSeq stranded mRNA according to the manufacturer’s instructions (Illumina Inc., San Diego, CA, USA). Libraries from *35Spro:RIF-RNAi* and their respective controls were sequenced on an Illumina HiSeq2000 platform, and those from *EXP2pro:RIF-RNAi, 35Spro:RIF* and their respective controls on a NextSeq550 platform, generating 100-bp single-end and 2 × 75-bp paired-end reads, respectively. Raw sequences were trimmed and mapped against using the assembly and annotation version v4.0.a1 of the *F. vesca* reference genome (https://www.rosacea.org/species/fragaria_vesca/genome_v4.0.a1;Edger et al., 2018), a high-quality reference genome, using CLC Genomics Workbench 9 (https://www.qiagenbioinformatics.com/products/clc-main-workbench/). MapMan bins were used for assignment of DEGs to functional categories (Usadel et al., 2009).

### Metabolome analysis

Primary and secondary metabolites were analyzed as described by Vallarino et al. (2018). For primary metabolites, the identification of metabolites was based on cross-reference with the Golm Database (Kopka et al., 2005). For secondary metabolites, putative identification and annotation were performed using literature, mainly from strawberry. Here, the data are presented as peak response in mass chromatograms and represented as a direct fraction of peak area. No quantitative standards were used in this study. ABA analysis by Gas Chromatography Time-Of-Flight Mass Spectrometry (GC–TOF–MS) was carried out according to Vallarino et al. (2019).

### Lignin staining

Lignified tissues were visualized using Weisner staining (Phloroglucinol-HCl, Sigma-Aldrich) (Clifford, 1974). This stain reacts with aldehyde groups in lignin, giving a characteristic deep reddish-purple coloration to the xylem in vascular bundles. In detail, fruits at the red stage were cut in slices and incubated in 1% phloroglucinol in a 70% (v/v) ethanol solution until they were totally cleared. Then the phloroglucinol solution was removed, and a few drops of 37% (v/v) HCl were added. The lignified tissues appeared with a pink-red coloration about 5 min later. Pictures were taken immediately since color faded in around 30 min.

### ABA treatment

Mock solution consisting of 2% (v/v) ethanol and 100 µM ABA (in 2% ethanol) were infiltrated in green fruits as previously described by Li et al. (2019). Five replicates per condition and genotype were used.

### Accession numbers

Sequence data from this article can be found in Supplemental Tables S3 and S4.

RNA-seq datasets were deposited at the Gene Expression Omnibus at NCBI under the accession number GSE167107.

## Supplemental Data

The following materials are available in the online version of this article.

Supplemental Figure S1. Phylogenetic analysis of NAC proteins.

Supplemental Figure S2 *.* Alignment of NAC proteins.

Supplemental Figure S3. Phenotype of ripe strawberries in control, and four independent stable *35Spro:RIF-*RNAi lines.

Supplemental Figure S4. Representative phenotypes of adult plants of the control and the different transgenic lines used in this work.

Supplemental Figure S5. Global transcriptome analysis in control and *35Spro:RIF-*RNAi receptacles.

Supplemental Figure S6. Alignment of *FaRIF* RNAi hairpin with *FaNAC042* homeologous sequences.

Supplemental Figure S7. MapMan enrichment analysis.

Supplemental Figure S8. Expression of cell-wall and aroma-related genes in receptacles of control and *35Spro:RIF*-RNAi fruits.

Supplemental Figure S9. Secondary and primary metabolism in receptacles of control and *35Spro:RIF*-RNAi fruits.

Supplemental Figure S10. Expression of *FaEXP2* at four ripening stages of receptacles and achenes.

Supplemental Data S1. DNA and protein sequence of *FaRIF/*FaRIF.

Supplemental Table S1. List of common DEGs in *35Spro:RIF-*RNAi #3 and #11.

Supplemental Table S2. List of oligonucleotides used in this study.

Supplemental Table S3. *F. vesca* ID numbers of genes mentioned in this work.

Supplemental Table S4. GenBank accession numbers.

Supplemental File 1. Alignment of the NAC proteins included in the phylogenetic tree.

Supplemental File 2. Newick format of the alignment shown in Supplemental File 1.

Supplemental Data Set S1. Transcriptome analysis in receptacles of control and *35Spro:RIF-*RNAi, *EXP2pro:RIF-*RNAi and *35Spro:RIF* lines.

Supplemental Data Set S2. List of common DEGs in the two *35Spro:RIF-*RNAi lines in both white and red stages of the receptacle ripening.

Supplemental Data Set S3. Expression data of differentially expressed *FaNAC* transcription factors and *NCED*s in control and *35Spro:RIF-*RNAi lines at white and red stages of receptacle ripening.

Supplemental Data Set S4. MapMan bins enrichment analysis.

Supplemental Data Set S5. Secondary metabolism analysis in control and *35Spro:RIF-*RNAi lines at three ripening stages.

Supplemental Data Set S6. Primary metabolism analysis in control and *35Spro:RIF-*RNAi lines at three ripening stages.

Supplemental Data Set S7. Summary of statistical analyses.

## Funding 

This work was supported by grants from the European Research Council (ERC-2014-StG 638134 to D.P.) and the Spanish Ministries of Economy and Competitiveness (MINECO, BIO2013-44199-R to V.V.) and of Science and Innovation (MICINN, RTI2018-09309-A-I00 to D.P., BIO2017-82609-R to M.A.B, and PID2019-111496RR-I00 to I.A.). D.P. and S.O. were supported by the Ramón y Cajal Programs RYC2013-12699 and RYC2011-09170, respectively (MINECO and MICINN, Spain). We thank Plan Propio from the University of Málaga for financial support, and Dr. José F. Sánchez Sevilla and the Departamento de Genómica y Biotecnología, IFAPA, Málaga, Spain to facilitate growing the transgenic plants at their facilities. We thank Patrice Salomé for his scientific editing and improvements to the manuscript.

*Conflict of interest statement*. The authors declare that they have no conflict of interest.

## Supplementary Material

koab070_Supplementary_Data

## Notes

C.M.-P. generated most constructs and transgenic plants and performed and analyzed most experiments and data. J.V., S.O. and J.G performed the RNA-seq experiment of the *35Spro:RIF-*RNAi lines. J.V., S.O, L.W. and A.R.F. were involved in the metabolome data acquisition and analysis. V.M., M.U., J.P. and I.A. contributed with plant *in vitro* maintenance and data analyses. A.C. and C.M. generated the *35Spro:*RNAi binary vector and performed the transformation of those constructs. C.M.-P. M.A.B., V.V. and D.P. designed the experiments. C.M.-P., V.V. and D.P. wrote the article. All authors commented on the article.

The author responsible for distribution of materials integral to the findings presented in this article in accordance with the policy described in the Instructions for Authors (https://academic.oup.com/plcell) is: David Posé (dpose@uma.es)
