# Supplementary material for: The NAC transcription factor FaRIF controls fruit ripening in strawberry
Source: Plant Cell. 2021 Feb 24;33(5):1574–93. doi: 10.1093/plcell/koab070 (PMC8254488; doi:10.1093/plcell/koab070)
Supplement: koab070_Supplementary_Data [file koab070_supplementary_data.zip › tpc.00833.2020-s01.pdf]

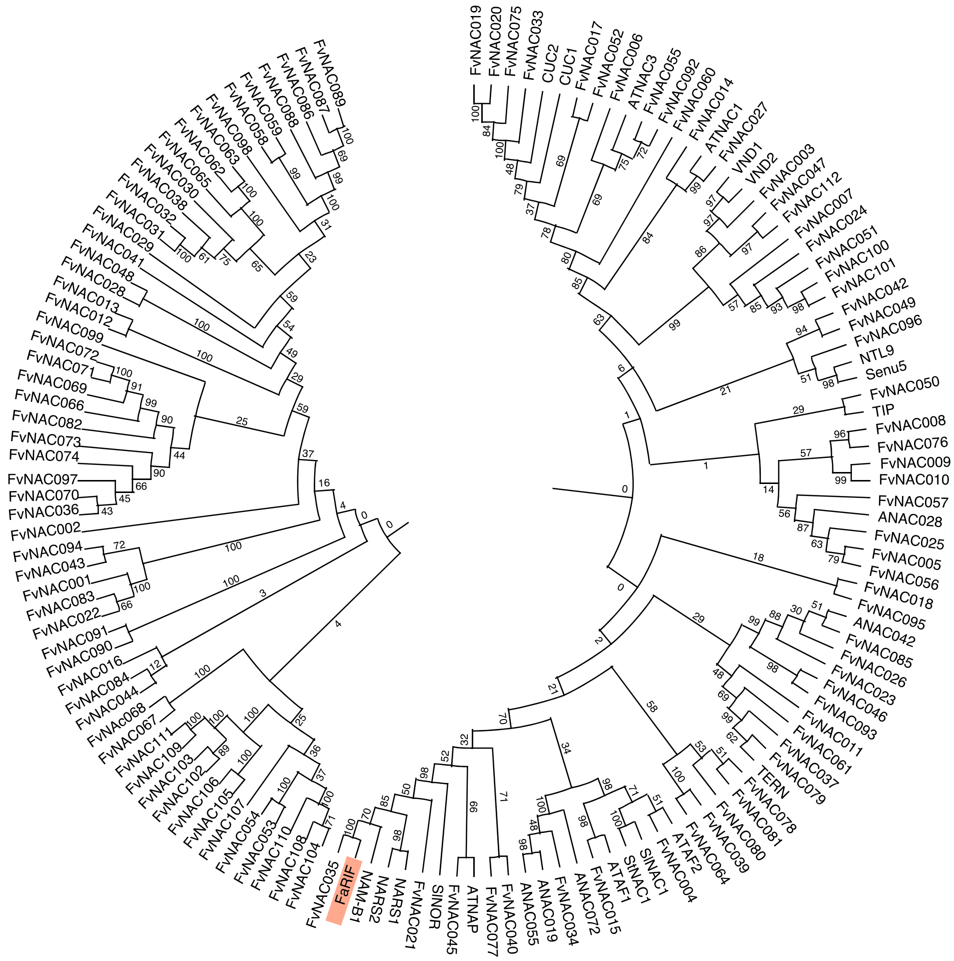

Supplemental Figure 1. Phylogenetic analysis of NAC proteins.
Neighbor-Joining analysis of FaNAC035 protein with the 112 NAC TFs identified in *F. vesca* and other NACs from other species. FaNAC035 is highlighted in pink. Numbers next to the nodes are bootstrap values from 1,000 pseudoreplicates. The protein sequences were obtained from GenBank (see Supplemental Table S4).
(Supports Figure 1).

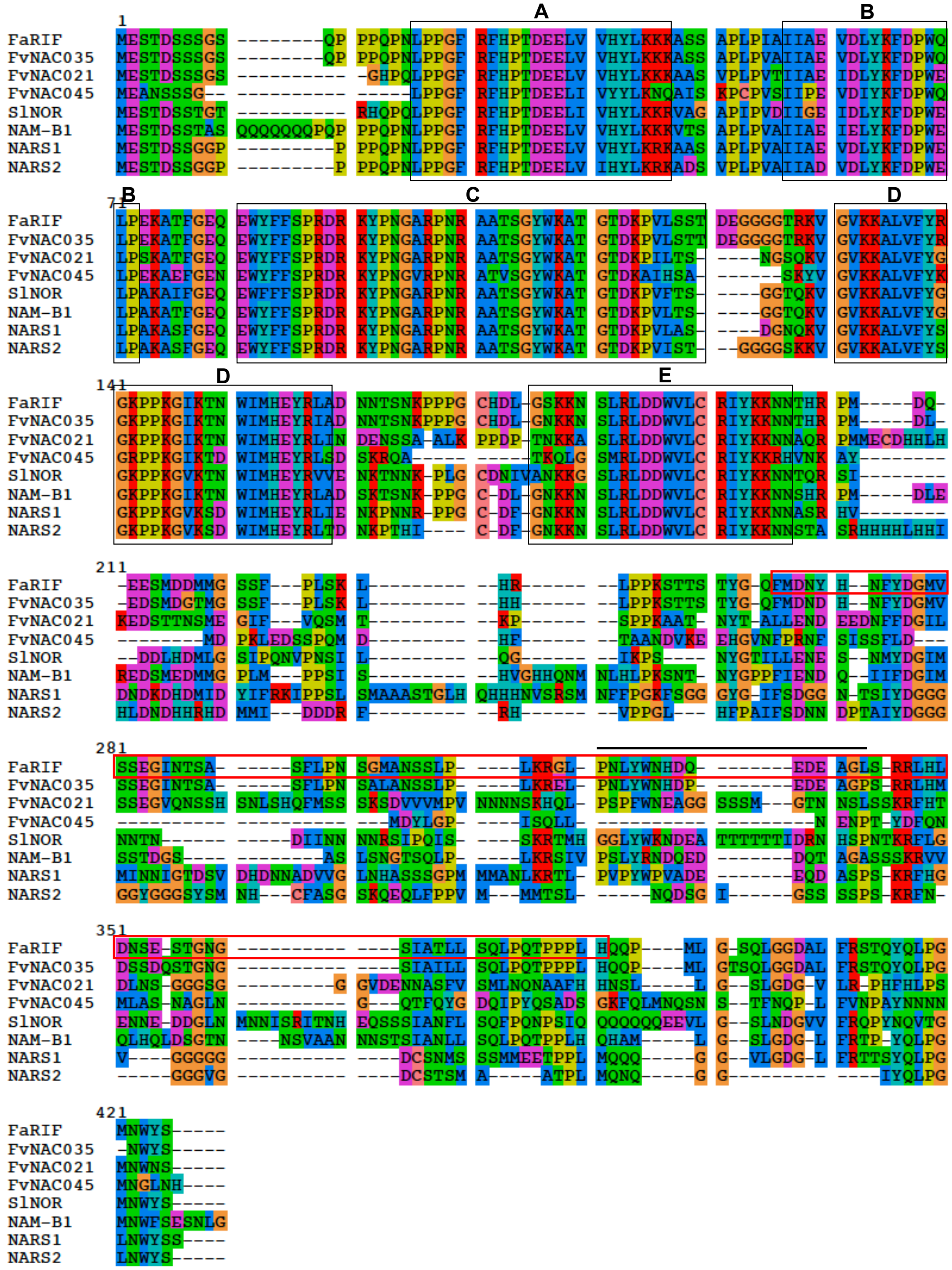

Supplemental Figure 2. Alignment of NAC proteins.

Proteins were selected based on the phylogenetic analysis (Supplemental Figure 1), all belonging to the same monophyletic group as FaRIF. FaRIF (*Fragaria* x *ananassa*), FvNAC035, FvNAC021 and FvNAC045 (*Fragaria vesca*), SlNOR (*Solanum lycopersicum*), NAM-B1 (*Prunus persica*), NARS1 and NARS2 (*Arabidopsis thaliana*). Black boxes indicate the five NAC domains (A-E). Red box indicates the region selected for the ihpRNA construct for *FaRIF* RNAi-mediated silencing. A black line indicates the peptide selected for the antiRIF antibody. All protein sequences were obtained from GenBank (Supplemental Data 2) and Moyano et al. (2018).
(Supports Figure 1).

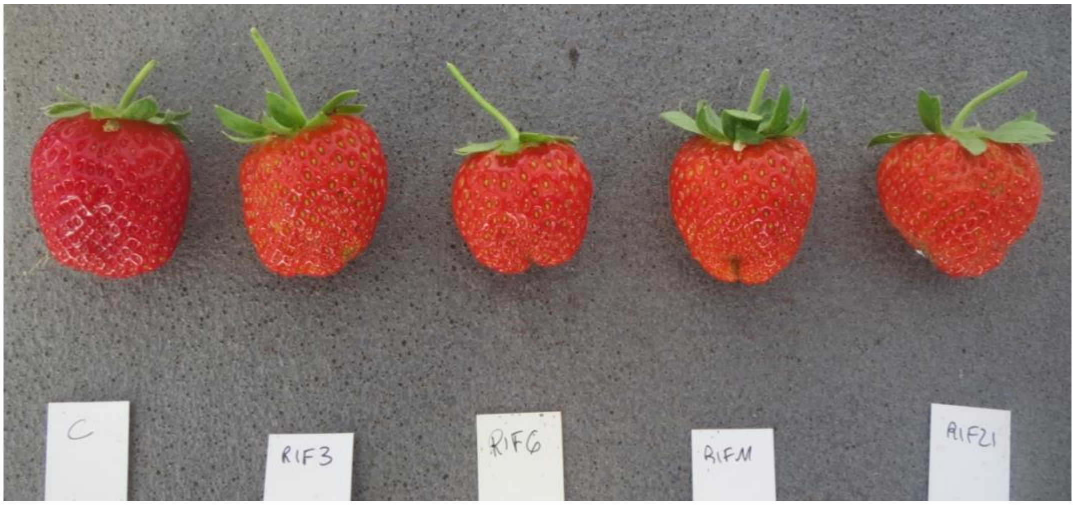

Supplemental Figure 3. Phenotype of ripe strawberries in control (C), and four independent stable $35S_{pro}$:*RIF*-RNAi lines (#3, #6, #11, and #21).
(Supports Figure 2).

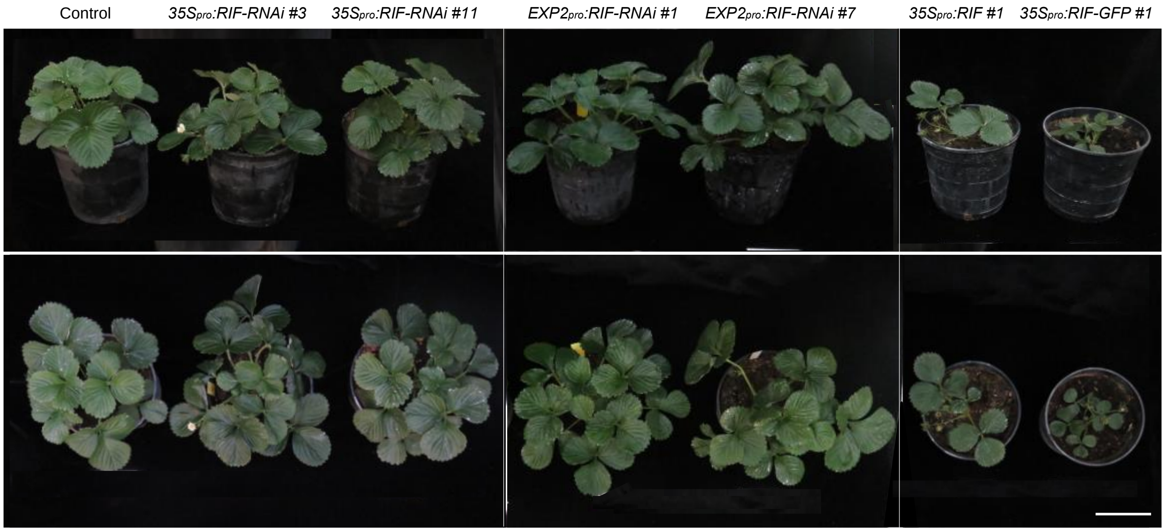

Supplemental Figure 4. Representative phenotypes of adult plants of the control and the different transgenic lines studied in this work. Scale bar, 10 cm.
(Supports Figures 2 and 6).

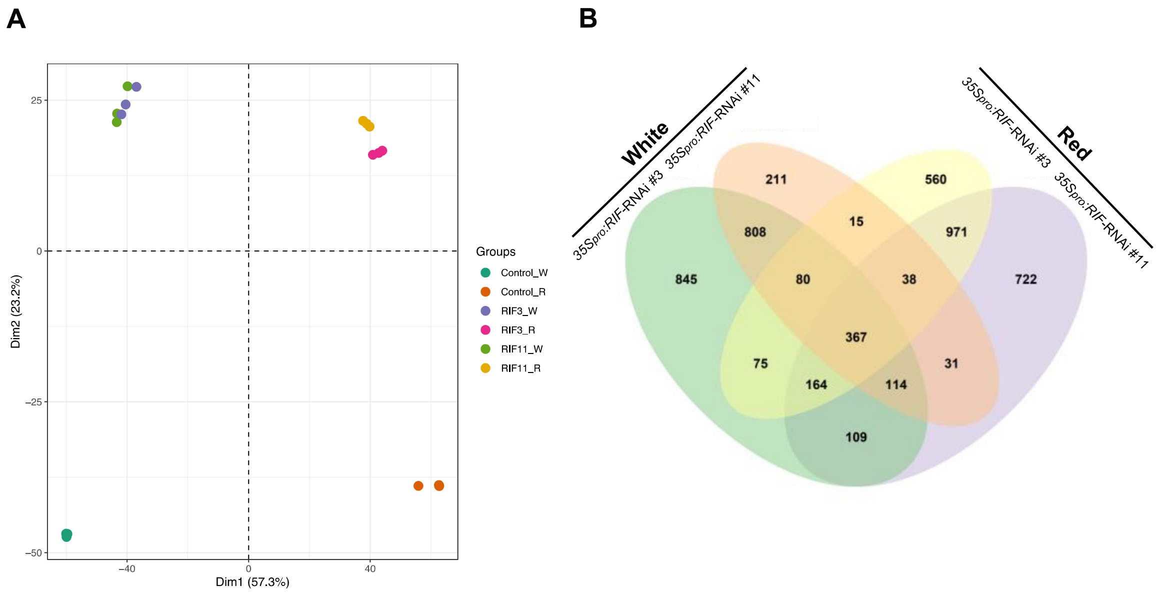

Supplemental Figure 5. Global transcriptome analysis in control and *35Spro:RIF*-RNAi receptacles.

A) Principal component analysis (PCA) for most variable genes (first quartile with the lowest p-value in ANOVA test) in white and red receptacles of control and *35Spro:RIF*-RNAi lines. C: control; RIF 3 and RIF 11: *35Spro:RIF*-RNAi #3 and #11, respectively; W: white stage; R: red stage.

B) Venn diagram including the DEGs at white and red receptacles from *35Spro:RIF*-RNAi #3 and #11 compared to the control (RPKM ≤ 1 at least in one of the samples and FDR ≤ 0,05).

(Supports Figures 3, 4 and 5).

```
FaRIF_RNAi                                    ------------------------------------------------------------        0
maker-Fvb4-3-augustus-gene-107.39-mRNA-1      ATGGAGAAGCTTAGTTTTGTGAGGAATGGAGAGCTGAGATTGCCTCCTGGTTTCCGGTTC        60
maker-Fvb4-2-snap-gene-75.53-mRNA-1           ATGGAGAAGCTTAGTTTTGTGAGGAATGGAGAGCTGAGATTGCCTCCTGGTTTTCGGTTC        60
maker-Fvb4-4-snap-gene-75.56-mRNA-1           ATGGAGAAGCTTAGTTTTGTGAGGAATGGAGAGCTGAGATTGCCTCCTGGTTTCCGGTTC        60


FaRIF_RNAi                                    ---------------------------------ATTC-----------------------        4
maker-Fvb4-3-augustus-gene-107.39-mRNA-1      CATCCAACTGACGAAGAGCTTGTTCTTCAGTACTTGAAGCGCAAGGTCTACTCCTGCCCT        120
maker-Fvb4-2-snap-gene-75.53-mRNA-1           CATCCAACTGATGAAGAGCTTGTTCTTCAGTACTTGAAGCGCAAGGTCTACTCCTGCCCT        120
maker-Fvb4-4-snap-gene-75.56-mRNA-1           CATCCAACTGACGAAGAGCTTGTTCTTCAGTACTTGAAGCGCAAGGTCTACTCCTGCCCT        120
                                                                               **

FaRIF_RNAi                                    ------------------------------------------------------------        4
maker-Fvb4-3-augustus-gene-107.39-mRNA-1      TTGCCTGCTTCCATCATCCCTGAGGTTGAGGTCTGTAAGTCTGATCCTTGGGATTTGCCA        180
maker-Fvb4-2-snap-gene-75.53-mRNA-1           TTGCCTGCTTCCATCATCCCTGAGGTTGAGGTCTGCAAGTCTGATCCTTGGGATTTGCCA        180
maker-Fvb4-4-snap-gene-75.56-mRNA-1           TTGCCCGCTTCCATCATCCCTGAGGTTGAGGTCTGCAAGTCTGATCCTTGGGATTTGCCA        180


FaRIF_RNAi                                    ------------------------------------------------------------        4
maker-Fvb4-3-augustus-gene-107.39-mRNA-1      GGTGACTTAGAGCAAGAGAGGTACTTCTTCAGCACTAGGGAGGCAAAGTATCCAAATGGG        240
maker-Fvb4-2-snap-gene-75.53-mRNA-1           GGTGACTTAGAGCAAGAGAGGTACTTCTTCAGCACTAGGGAGGCAAAGTACCCAAATGGG        240
maker-Fvb4-4-snap-gene-75.56-mRNA-1           GGTGACTTAGAGCAAGAGAGGTACTTCTTCAGCACTAGGGAGGCAAAGTACCCAAATGGG        240


FaRIF_RNAi                                    ------------------------------------------------------------        4
maker-Fvb4-3-augustus-gene-107.39-mRNA-1      AACAGATCAAACAGAGCTACAGGTTCTGGGTATTGGAAGGCAACTGGTTTGGACAAGCAA        300
maker-Fvb4-2-snap-gene-75.53-mRNA-1           AACAGATCAAACAGAGCTACAGGTTCTGGGTATTGGAAGGCAACTGGTTTGGACAAGCAA        300
maker-Fvb4-4-snap-gene-75.56-mRNA-1           AACAGATCAAACAGAGCTACAGGTTCTGGGTATTGGAAGGCAACTGGTTTGGACAAGCAA        300


FaRIF_RNAi                                    ---------------------------------ATGGACAA----------------CGA        15
maker-Fvb4-3-augustus-gene-107.39-mRNA-1      ATTGTGGCTTCCAGGGGTAACCAAGTTGTGGGGATGAAGAAAACTTTGGTTTTTTACAGA        360
maker-Fvb4-2-snap-gene-75.53-mRNA-1           ATTGTGGCTTCTAGGGGTAACCAAGTTGTGGGGATGAAGAAGACTCTGGTTTTTTACAGA        360
maker-Fvb4-4-snap-gene-75.56-mRNA-1           ATTGTGGCTTCTAGGGGTAACCAAGTTGTGGGGATGAAGAAGACTCTGGTTTTTTACAGA        360
                                                                               *** * **                 **

FaRIF_RNAi                                    CCACAATTTCTACGACGGGATGGTAAGCAGCGAAGGGATTAATACTAGTGCTTCTTTTCT        75
maker-Fvb4-3-augustus-gene-107.39-mRNA-1      GGTAAACCTCCACATGGGGCTCGGACCGATTGGATTATGCACGAGTATCGCCTCGTTTTA        420
maker-Fvb4-2-snap-gene-75.53-mRNA-1           GGTAAACCTCCACATGGGGCTAGGACCGATTGGATTATGCACGAGTATCGCCTCGTTTTA        420
maker-Fvb4-4-snap-gene-75.56-mRNA-1           GGTAAACCTCCACATGGGGCTAGGACCGATTGGGTTATGCACGAGTATCGCCTCGTTTTA        420
                                                  **  ** **   *** * * *   *  *        *  * **  ** ** ***

FaRIF_RNAi                                    TCCAAACTCGGGT--------ATGGCCAACAGATCTCTCCCTCTGAAACGGGGA------        121
maker-Fvb4-3-augustus-gene-107.39-mRNA-1      GCTGAAGATAAAAATAACTCCACTACCCAAAGCCATGTTCCGGTGAACAATTGGGTTCTT        480
maker-Fvb4-2-snap-gene-75.53-mRNA-1           GCTGAAGATAAAAATAACTCCACTACCCAAAGCCATGTTCCGGTGAACAATTGGGTTCTT        480
maker-Fvb4-4-snap-gene-75.56-mRNA-1           GCTGAAGATAAAAATAACTCCACTACCCAAAGCCATGTTCCGGTGAACAATTGGCTTCTT        480
                                               *  **               *   ** * **   * * **  ****     *

FaRIF_RNAi                                    ---------CTCCCGAATCTGTACTGGAATCATGATCAGGAGGACGAA------GCAGGG        166
maker-Fvb4-3-augustus-gene-107.39-mRNA-1      TGCCGCATATTTTTGAAGAAAAGAGGCAGTGGTAAAAATGAAGAGGAACAAGTTCAAGTG        540
maker-Fvb4-2-snap-gene-75.53-mRNA-1           TGCCGCATATTTTTGAAGAAAAGAGGCAGTGGTAAAAATGAGGAGGAACAAGTTCAAGGG        540
maker-Fvb4-4-snap-gene-75.56-mRNA-1           TGCCGCATATTTTTGAAGAAAAGAGGCAGTGGTAAAAATGAGGAGGAACAAGTTCAAGGG        540
                                                        *   ***        * * *  * *  * ** ** ***       ** *

FaRIF_RNAi                                    CTTTCAAGGAGACTACACCTAGACAACAGTGAGAGCACCGGAAATGGTTCTATTGCAACT        226
maker-Fvb4-3-augustus-gene-107.39-mRNA-1      CAGGCCTGCAATGTTGACCGAG----TGGCGAAAAAACCAAGGATTACTCGACCTGTTTT        596
maker-Fvb4-2-snap-gene-75.53-mRNA-1           CAGGCCTGCAATGTTGACCGAG----TGGTGAAAAAACCAAGGATTACTCGGCCTGCTTT        596
maker-Fvb4-4-snap-gene-75.56-mRNA-1           CAGGCCTGCAATGTTGACCGAG----TGGTGAAAAAACCAAGGATTACTCGGCCTGTTTT        596
                                              *   *  * *   * *** **      * ** *  ***    **   **         *

FaRIF_RNAi                                    CTGCTTTCTCAG-----CTCCCTCAGACACCTCCTCCATTGCAC----------------        265
maker-Fvb4-3-augustus-gene-107.39-mRNA-1      CTACGATTTCATGACCAAAGACAGAGCCAAATTGAGCCTTGCGCCTTGTTCTTCATCCTC        656
maker-Fvb4-2-snap-gene-75.53-mRNA-1           CTACGATTTCATGACCAAAGACAGAGCCAACTTGAGCCTTGCGCCTTGTTCTTCATCCTC        656
maker-Fvb4-4-snap-gene-75.56-mRNA-1           CTACGATTTCATGACCAAAGACAGAGCCAACTTGAGCCTTGCGCCTTGTTCTTCATCCTC        656
                                              ** *  * ***          *  ** **  *    * **** *

FaRIF_RNAi                                    ------------------------------------------------------------        265
maker-Fvb4-3-augustus-gene-107.39-mRNA-1      AGGTTCAAGTGGAGTCACAGATGTCGTATCTAGCGAGACAGAGGAGCGTGAAGAGAGCAG        716
maker-Fvb4-2-snap-gene-75.53-mRNA-1           AGGTTCAAGTGGAGTCACAGATGTCGTATCTAGCGAGACAGAGGAGCATGAAGAGAGCAG        716
maker-Fvb4-4-snap-gene-75.56-mRNA-1           AGGTTCAAGTGGAGTCACAGATGTCGTATCTAGCGAGACAGAGGAGCATGAAGAGAGCAG        716


FaRIF_RNAi                                    ----------------------------------------------------- 265
maker-Fvb4-3-augustus-gene-107.39-mRNA-1      TAGCTGCAATAGTCTTTCTTATTTTAGAAGAAAACAGTGA------------- 756
maker-Fvb4-2-snap-gene-75.53-mRNA-1           TAGCTGCAATAGTCTTTCTTATTTTAGAAGAAAACAGTGA------------- 756
maker-Fvb4-4-snap-gene-75.56-mRNA-1           TAGCTGCAATAGTCTTCTTATTTTAGAAGA-AAACAGTGAAGTCTCCTTCTGA 768
```

Supplemental Figure 6. Alignment of *FaRIF* RNAi hairpin with *FaNAC042* homeologous sequences. (Supports Figure 3).

A

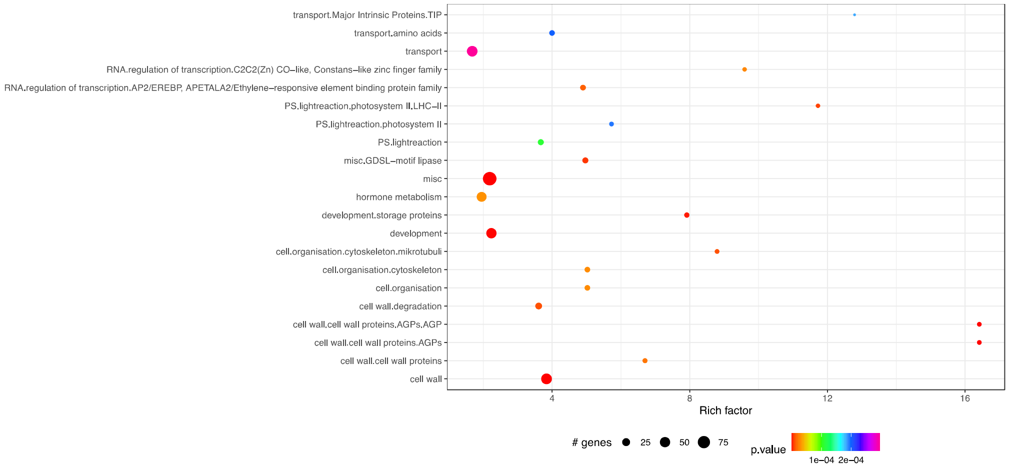

B

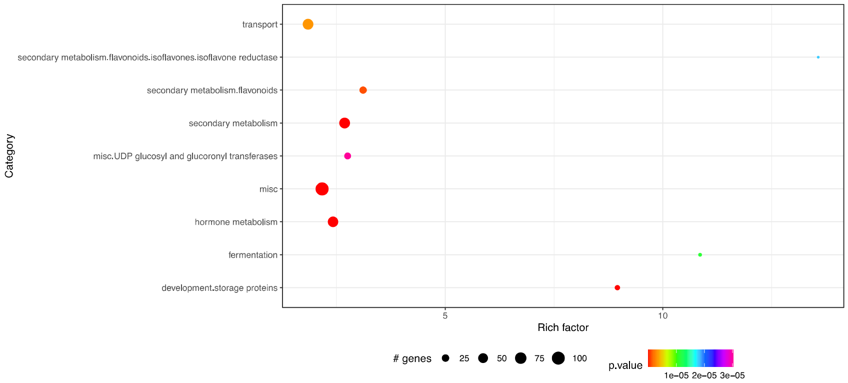

Supplemental Figure 7. MapMan enrichment analysis.
MapMan bin categories of DEGs in *35Spro:RIF*-RNAi receptacles (RPKM ≤ 1 at least in one of the samples, FDR ≤ 0,05, and ≥ 2-fold up- or downregulation in the RNAi lines). Significantly enriched categories (p-value ≤ 0,05) are shown for white **(A)** and red **(B)** stages. Categories contained in a more general category included using this threshold were removed from the plots.
(Supports Figures 3, 4 and 5).

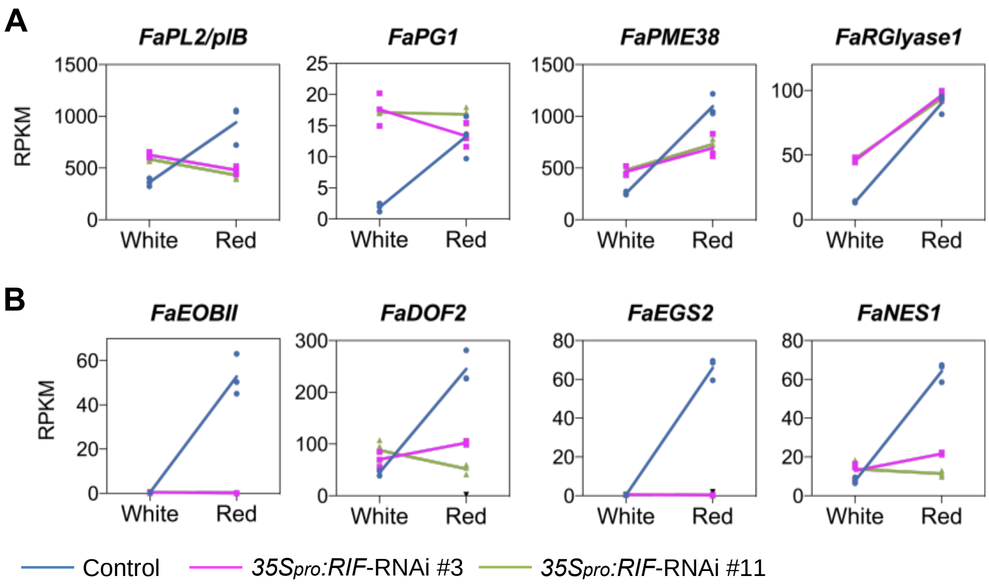

Supplemental Figure 8. Expression of cell wall- **(A)** and aroma-related genes **(B)** in receptacles of control and *35Spro:RIF*-RNAi fruits.
(Supports Figure 3).

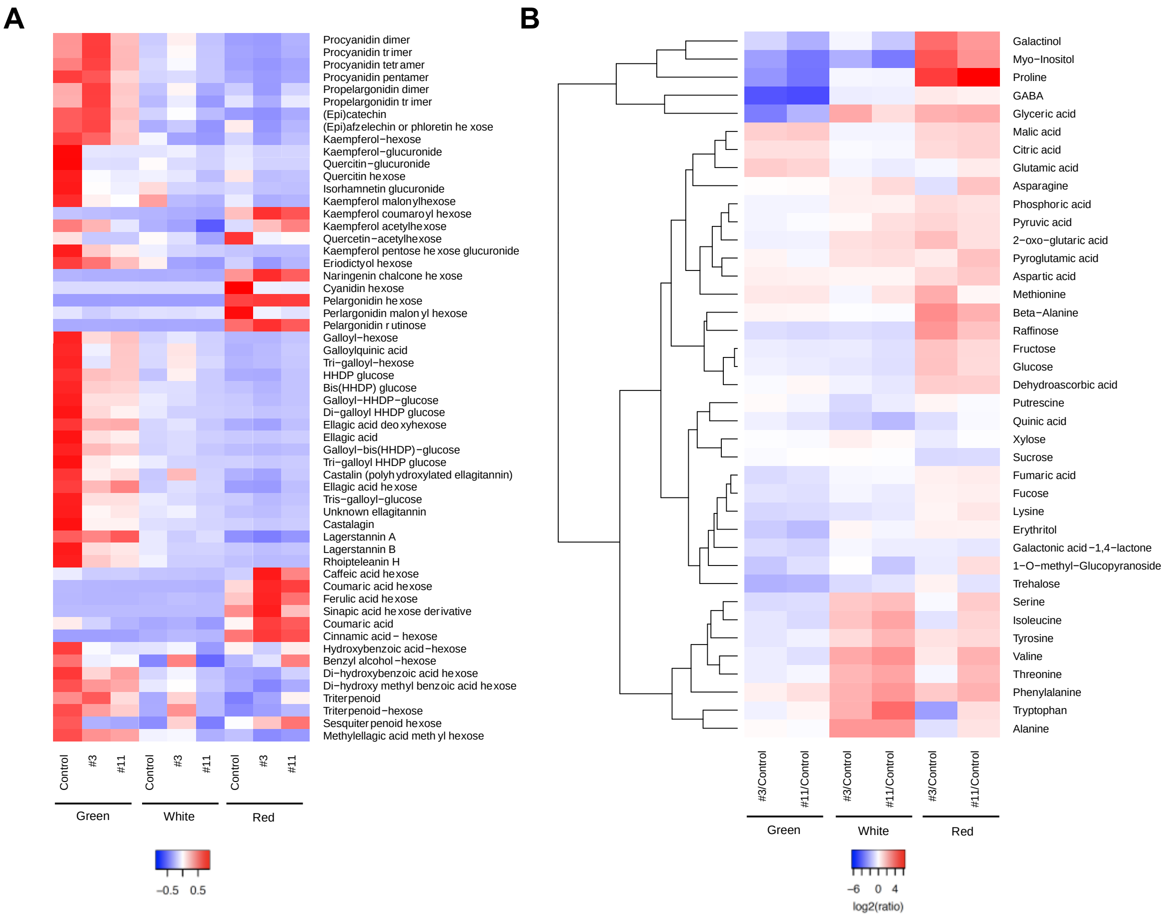

Supplemental Figure 9. Secondary and primary metabolism in receptacles of control and *35Spro:RIF*-RNAi fruits.

A) Heat map of secondary metabolism during strawberry ripening in control and *35Spro:RIF*-RNAi receptacles. A color-coded matrix represents range-scaled mean values of the metabolite intensity.

B) Hierarchical clustering analysis representing the ratio of the levels of primary metabolites. Data are normalized to mean response (n = 3). The scale is logarithmic (log_2). Values are displayed in false color code.

(Supports Figures 3 and 5).

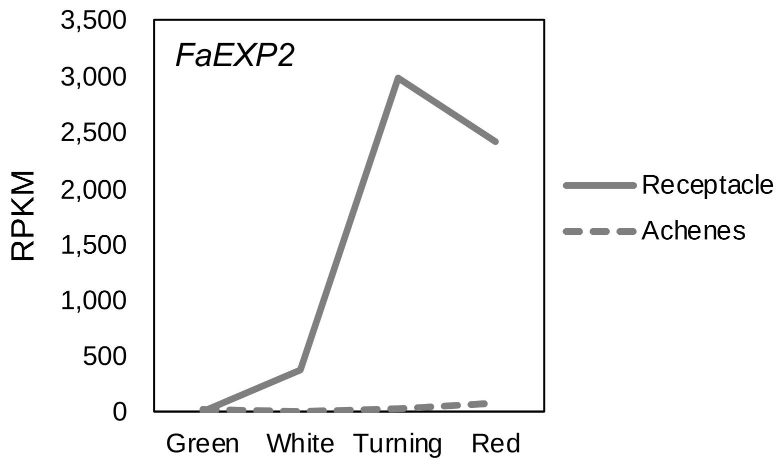

Supplemental Figure 10. Expression of *FaEXP2* at four ripening stages of receptacles and achenes. Data from Sánchez-Sevilla et al. (2017).
(Supports Figure 6).

Supplemental Data 1. DNA and protein sequence of *FaRIF*/FaRIF*.* DNA sequence selected for the ihpRNA construct for the *FaRIF* RNAi-mediated silencing is marked in red. Peptide sequence selected for the anti-RIF antibody is marked in blue.

>*FaRIF* CDS
ATGGAGAGCACCGACTCGTCTTCCGGCTCGCAGCCGCCGCCGCAGCCAAACCTACCGCC
GGGATTCCGCTTCCACCCCACCGATGAGGAGCTAGTCGTTCATTACCTCAAGAAAAAGG
CCTCCTCGGCTCCCCTCCCCATTGCCATCATCGCCGAAGTCGACCTCTACAAATTTGAT
CCATGGCAGCTCCCAGAAAAGGCGACGTTCGGAGAGCAAGAGTGGTATTTTTTCAGTCC
TAGAGACCGGAAGTACCCGAACGGAGCACGGCCTAATAGAGCAGCGACTTCAGGATATT
GGAAGGCGACCGGAACTGACAAGCCCGTTTTGAGTAGTACTGATGAGGGAGGTGGGGGT
ACTCGGAAAGTTGGGGTGAAAAAGGCACTTGTTTTCTACAGAGGAAAGCCCCCAAAAGG
AATCAAAACCAATTGGATCATGCATGAGTATAGGCTTGCTGATAACAACACAAGTAACA
AGCCACCACCTGGGTGTCATGACTTGGGTAGCAAGAAGAACTCCTTAAGGCTTGATGAT
TGGGTGCTTTGTCGAATTTACAAGAAGAACAACACGCATAGGCCGATGGATCAGGAGGA
ATCCATGGACGACATGATGGGATCGTCGTTCCCACTGTCGAAGCTGCACCGCCTTCCCC
CGAAATCGACAACATCAACCTATGGCCAATTCATGGACAACTACCACAATTTCTACGAC
GGGATGGTAAGCAGCGAAGGGATTAATACTAGTGCTTCTTTTCTTCCAAACTCGGGTAT
GGCCAACAGCTCTCTCCCCCTAAAACGGGGACTCCCGAATCTGTACTGGAATCATGATC
AGGAGGACGAAGCAGGGCTTTCAAGGAGACTACACCTGGACAACAGTGAGAGCACTGGA
AATGGTTCTATTGCAACTCTGCTTTCTCAGCTCCCTCAGACACCTCCTCCATTGCACCA
GCAGCCAATGCTGGGGTCACAGCTCGGTGGCGACGCTCTGTTTCGTAGTACACAGTATC
AACTTCCGGGGATGAATTGGTATTCTTAG

>FaRIF Protein sequence
MESTDSSSGSQPPPQPNLPPGFRFHPTDEELVVHYLKKKASSAPLPIAIIAEVDLYKFD
PWQLPEKATFGEQEWYFFSPRDRKYPNGARPNRAATSGYWKATGTDKPVLSSTDEGGGG
TRKVGVKKALVFYRGKPPKGIKTNWIMHEYRLADNNTSNKPPPGCHDLGSKKNSLRLDD
WVLCRIYKKNNTHRPMDQEESMDDMMGSSFPLSKLHRLPPKSTTSTYGQFMDNYHNFYD
GMVSSEGINTSASFLPNSGMANSSLPLKRGLPNLYWNHDQEDEAGLSRRLHLDNSESTG
NGSIATLLSQLPQTPPPLHQQPMLGSQLGGDALFRSTQYQLPGMNWYS*

Supplemental Table 1. List of common DEGs in *35Spro:RIF*-RNAi #3 and #11.

GENE	*F. vesca* IDs	Mean log_2 fold-change WHITE	Mean log_2 fold-change RED	References
**PHYTOHORMONES**				
**Metabolism**				
NCED3	FvH4_3g16730	–7.5	–8.6	(Jia et al., 2011)
CYP79B	FvH4_3g09210	Induced	51.2	(Sugawara et al., 2009)
SAMDC1	FvH4_6g45760	–2.1	–1.5	(Guo et al., 2018)
SAMDC2	FvH4_4g28650	–3.8	–3.9	(Guo et al., 2018)
**Signaling**				
HVA22	FvH4_5g08670	–3.9	–3.0	(Shen et al., 2001)
SnRK2.6	FvH4_2g06910	2.6	2.9	(Han et al., 2015)
AP2/AIL6	FvH4_1g19530	6.2	11.5	(Krizek et al., 2016)
IAA9	FvH4_1g18900	–1.9	–3.2	(Wang et al., 2005)
SHP	FvH4_6g37880	3.0	1,7	(Daminato et al., 2013)
ASR	FvH4_2g13410	2.3	2.7	(Jia et al., 2016)
NAC022	FvH4_3g08490	2.9	–1.4	(Carrasco-Orellana et al., 2018)
ETR2	FvH4_4g03030	1.8	2.0	(Merchante et al., 2013)
EIN3b	FvH4_7g04940	1.6	1.5	(Merchante et al., 2013)
ERF99a	FvH4_7g26920	7.8	2.9	(Sánchez-Sevilla et al., 2017)
ERF17/HRE2	FvH4_2g26080	16.8	7.2	(Sánchez-Sevilla et al., 2017)
ERF74/RAP2-12	FvH4_6g35670	1.4	1.3	(Giuntoli et al., 2017)
**DEVELOPMENT**				
**Regulatory**				
HY5	FvH4_2g29440	–3.5	–3.3	(Gangappa and Botto, 2016)
BBX19	FvH4_1g12110	–7.7	–6.9	(Gangappa and Botto, 2014)
**Carbohydrate Metabolism**				
SUS1	FvH4_2g26000	10.7	4.3	(Luo et al., 2020)
PFK6	FvH4_2g35190	2.7	2.4	(Mustroph et al., 2014)
FBA8/Aldolase	FvH4_6g17970	1.7	1.7	(van der Linde et al., 2011)
PDC	FvH4_3g08040	3.5	3.4	(Kürsteiner et al., 2003)
ADH	FvH4_2g14750	7.3	4.3	(Ismond et al., 2003)
ADH	FvH4_2g14760	13.1	17.0	(Ismond et al., 2003)
PEPCK	FvH4_6g02090	–12.0	–6.4	(Fontaine et al., 2002)

Supplemental Table 2. List of oligonucleotides used in this study.

Purpose	Oligo	Sequence (5' -> 3')
FaRIF cloning	P13	ATGGAGAGCACCGACTCGTC
	P14	CGAAATTGGGCTAAGAATACCAAT
pCM1 (Expansin promoter)	P1	GAGCTCCAAGTAATATTATCTCTTGAA
	P2	TGTCCATGAATCTCGAGCGGGGTAGAAGGAGAAGAAGGTG
pCM1 (Expansin promoter)	P1	GAGCTCCAAGTAATATTATCTCTTGAA
	P11	TGTCCATGAATGAATTCCGGGGTAGAAGGAGAAGAAGGTG
RIF-RNAi-PDK intron	P4	TCGATTTCGAACCCAATTTC
	P12	TCTCCTTCTACCCCGGAATTCATTCATGGACAACGACCAC
pCM2	P1	GAGCTCCAAGTAATATTATCTCTTGAA
	P4	TCGATTTCGAACCCAATTTC
pCM23 (*35S_{pro}:RIF*)	P8	ACCGGATCCAGATCCAGATCCAGAATACCAATTCATCCC
	P13	ATGGAGAGCACCGACTCGTC
GFP	P9	TCTGGATCTGGATCCGGTTCTGCGGCCGACAGTAAAGGAGAAGAA
	P10	CTCGAGTTAGTGGTGGTGGTGGTGGTGTTTG
pCM10 (fusion *RIF-GFP*)	P13	ATGGAGAGCACCGACTCGTC
	P10	CTCGAGTTAGTGGTGGTGGTGGTGGTGTTTG
35S_{pro}:RIF-RNAi	P649	GGATCCCTCGAGATTCATGGACAACGACCACA
	P650	ATCGATGGTACCGTGCAATGGAGGAGGTGTCT
qPCR *FaRIF*	P651	GAGCACCGGAAATGGTTCTA
	P652	CCCCGGAAGTTGATACTGTG
qPCR CHP1 (housekeeping)	FaCHP1-F	TGCATATATCAAGCAACTTTACACTGA
	FaCHP1-R	ATAGCTGAGATGGATCTTCCTGTGA

Supplemental Table 3. *F. vesca* ID numbers of genes mentioned in this work.

**Gene**	***F.vesca* ID**
FaRIF (*FaNAC035*)	FvH4_3g20700
FaMYB10	FvH4_1g22020
FaMYB1	FvH4_5g17120
FaGAMYB	FvH4_7g04470
MYB44.2	FvH4_2g33810
FaEOBII	FvH4_6g50930
FaDOF2	FvH4_2g14390
FaEGS2	FvH4_2g09110
FaSPT	FvH4_1g16230
FaPRE1	FvH4_3g04290
FaSHP	FvH4_6g37880
FaMADS9	FvH4_6g46420
NAC006	FvH4_1g27900
NAC010	FvH4_2g13330
NAC015	FvH4_2g27430
NAC021	FvH4_3g04630
NAC022 (*FcNAC1*)	FvH4_3g08490
NAC033	FvH4_3g19410
NAC034	FvH4_3g20690
NAC042	FvH4_4g23130
NAC096	FvH4_7g06500
FaXYL3	FvH4_5g37840
FaPL3	FvH4_5g06720
FaPL4	FvH4_4g25110
FaGH9B15	FvH4_5g03220
FaADPG2	FvH4_2g20970
FaEXP1	FvH4_6g13610
FaEXP2	FvH4_7g25860
FaEXP3	FvH4_3g36410
FaPME39	FvH4_6g35830
*AGP*s	FvH4_1g03420
	FvH4_3g26750
	FvH4_3g29120
	FvH4_3g36000
	FvH4_6g20860
	FvH4_6g40590
	FvH4_7g12120
FaPL2/plB	FvH4_2g19540
FaPG1	FvH4_6g41380
FaPME38	FvH4_6g35820
FaRGlyase1	FvH4_1g03300
FaNES1	FvH4_3g03150
PAL1	FvH4_6g16060
PAL2	FvH4_7g19130
C4H	FvH4_3g40570
4CL2	FvH4_7g33990
CHS	FvH4_7g01160
CHI1	FvH4_7g25890
CHI2	FvH4_7g20870
F3H	FvH4_1g11810

Supplemental Table 3 (continued). *F. vesca* ID numbers of genes mentioned in this work.

Gene	***F.vesca*** **ID**
DFR1	FvH4_2g39530
DFR2	FvH4_2g39520
F3'H	FvH4_5g14010
LARs	FvH4_4g28110
	FvH4_5g04260
ANS	FvH4_5g01170
ANR1	FvH4_3g02980
ANR2	FvH4_2g09120
UFGT1	FvH4_1g19400
UFGT2	FvH4_7g15020
HCT	FvH4_6g28410
C3H	FvH4_1g29330
COMT	FvH4_2g05780
CCR	FvH4_6g28680
CAD9	FvH4_1g23790
PRXs	FvH4_1g17340
	FvH4_7g33030
	FvH4_6g10250
	FvH4_2g13110
FaNCED3	FvH4_3g16730
FaNCED5 / FaNCED2	FvH4_3g05440
FaZEP	FvH4_1g16080
FaHVA22	FvH4_5g08670
FaSnRK2.6	FvH4_2g06910
CYP79B	FvH4_3g09210
AIL6	FvH4_1g19530
FaIAA9	FvH4_1g18900
FaASR	FvH4_2g13410
ETR2	FvH4_4g03030
EIN3b	FvH4_7g04940
FaHY5	FvH4_2g29440
FaBBX19	FvH4_1g12110
FaSUS1	FvH4_2g26000
FaSPS1	FvH4_2g28820
FaPFK6	FvH4_2g35190
FaFBA8	FvH4_6g17980
FaPDC1	FvH4_3g08040
FaADHs	FvH4_2g14750
	FvH4_2g14760
FaERF-74/RAP2.12	FvH4_6g35670
FaERF-17/HRE2	FvH4_2g26080
FaPEPC	FvH4_3g18430
FaPEPCK	FvH4_6g02090
FaPDHK	FvH4_4g21480
FaNADHDH	FvH4_1g30070
FaPK	FvH4_4g37360
FaPDH	FvH4_3g05390
FaCS	FvH4_4g21220
FaGAD	FvH4_1g30330
FaGABA-T	FvH4_6g39530
PDHE1α	FvH4_3g05390

Supplemental Table 4. GenBank accession numbers.

Species	Gene name	GenBank ID
Arabidopsis thaliana	*ANAC019*	NP_175697.1
	ANAC028	NP_176766.1
	ANAC042	NP_181828.1
	ANAC055	NP_188169
	ANAC072	NP_001078452
	ATAF1	NP_171677
	ATAF2	NP_680161
	AtNAC1	AAF21437
	AtNAC3	AAP42729
	AtNAP	NP_564966.1
	CUC1	BAB20598.1
	CUC2	BAA19529
	NARS1	NP_188170.1
	NARS2	NP_175696.1
	VND1	NP_179397.1
	VND2	NP_195339.1
	NTL9	NP_001119122.1
Nicotiana tabacum	*TERN*	BAA78417.1
Petunia × hybrida	*TIP*	AAM47025.1
Solanum lycopersicum	*SlNAC1*	NP_001234482.1
	Senu5	CAA99760
	SlNOR	Solyc10g006880.2.1
Solanum tuberosum	*StNAC1*	NP_001305595.1
Prunus persica	*NAM-B1*	CAG28971

SlNOR sequence was obtained from the Solanaceae database (https://solgenomics.net). All the sequences from NAC proteins in *F. vesca* were obtained from Moyano et al. (2018).

Genetics Materials Module.

Transgenic line	Description
35Spro:RIF-RNAi #3	T1 RNAi transgenic line generated in *F.* × *ananassa* cv. Camarosa. Together with line #11, it was the line selected for deep characterization.
35Spro:RIF-RNAi #6	T1 RNAi transgenic line generated in *F.* × *ananassa* cv. Camarosa. It showed the same fruit and ripening phenotype than the other three *35Spro*-RNAi lines, but it was not selected for a deeper characterization.
35Spro:RIF-RNAi #11	T1 RNAi transgenic line generated in *F.* × *ananassa* cv. Camarosa. Together with line #3, it was the line selected for deep characterization.
35Spro:RIF-RNAi #21	T1 RNAi transgenic line generated in *F.* × *ananassa* cv. Camarosa. As with line #6, it showed the same fruit and ripening phenotype than the other three *35Spro*-RNAi lines, but it was not selected for a deeper characterization.
EXP2pro:RIF-RNAi #1	T1 RNAi transgenic line generated in *F.* × *ananassa* cv. Camarosa. Together with line #7, it was the line selected for deep characterization
EXP2pro:RIF-RNAi #4	T1 RNAi transgenic line generated in *F.* × *ananassa* cv. Camarosa. It showed the same fruit phenotype than the other four *EXP2pro*-RNAi lines, but it was not selected for characterization.
EXP2pro:RIF-RNAi #7	T1 RNAi transgenic line generated in *F.* × *ananassa* cv. Camarosa. Together with line #1, it was the line selected for deep characterization.
EXP2pro:RIF-RNAi #10	T1 RNAi transgenic line generated in *F.* × *ananassa* cv. Camarosa. As line #4, it showed the same fruit phenotype than the other four *EXP2pro*-RNAi lines, but it was not selected for characterization.
35Spro:RIF #1	T1 overexpression transgenic line generated in *F.* × *ananassa* cv. Camarosa. It was the only surviving line that overexpressed *FaRIF*, and therefore, selected for characterization.
35Spro:RIF-GFP #1	T1 overexpression transgenic line generated in *F.* × *ananassa* cv. Camarosa. It was the only surviving line that overexpressed *FaRIF*, in this case fused to GFP, and therefore, selected for characterization.

References

Carrasco-Orellana, C., Stappung, Y., Mendez-Yañez, A., Allan, A.C., Espley, R.V., Plunkett, B.J., Moya-Leon, M.A., and Herrera, R. (2018). Characterization of a ripening-related transcription factor FcNAC1 from *Fragaria chiloensis* fruit. Sci Rep. **8**: 10524.

Daminato, M., Guzzo, F., and Casadoro, G. (2013). A *SHATTERPROOF-like* gene controls ripening in non-climacteric strawberries, and auxin and abscisic acid antagonistically affect its expression. J. Exp. Bot. **64**: 3775–3786.

Fontaine, V., Hartwell, J., Jenkins, G.I., and Nimmo, H.G. (2002). *Arabidopsis thaliana* contains two phosphoenolpyruvate carboxylase kinase genes with different expression patterns. Plant, Cell & Environment **25**: 115–122.

Gangappa, S.N. and Botto, J.F. (2014). The BBX family of plant transcription factors. Trends Plant Sci. **19**: 460–470.

Gangappa, S.N. and Botto, J.F. (2016). The Multifaceted Roles of HY5 in Plant Growth and Development. Mol Plant **9**: 1353–1365.

Giuntoli, B., Shukla, V., Maggiorelli, F., Giorgi, F.M., Lombardi, L., Perata, P., and Licausi, F. (2017). Age-dependent regulation of ERF-VII transcription factor activity in *Arabidopsis thaliana*. Plant, Cell & Environment **40**: 2333– 2346.

Guo, J., Wang, S., Yu, X., Dong, R., Li, Y., Mei, X., and Shen, Y. (2018). Polyamines Regulate Strawberry Fruit Ripening by Abscisic Acid, Auxin, and Ethylene. Plant Physiology **177**: 339–351.

Han, Y., Dang, R., Li, J., Jiang, J., Zhang, N., Jia, M., Wei, L., Li, Z., Li, B., and Jia, W. (2015). SUCROSE NONFERMENTING1-RELATED PROTEIN KINASE2.6, an ortholog of OPEN STOMATA1, is a negative regulator of strawberry fruit development and ripening. Plant Physiology **167**: 915–930.

Ismond, K.P., Dolferus, R., de Pauw, M., Dennis, E.S., and Good, A.G. (2003). Enhanced low oxygen survival in Arabidopsis through increased metabolic flux in the fermentative pathway. Plant Physiology **132**: 1292–1302.

Jia, H., Jiu, S., Zhang, C., Wang, C., Tariq, P., Liu, Z., Wang, B., Cui, L., and Fang, J. (2016). Abscisic acid and sucrose regulate tomato and strawberry fruit ripening through the abscisic acid-stress-ripening transcription factor. Plant Biotechnol. J. **14**: 2045–2065.

Jia, H.-F., Chai, Y.-M., Li, C.-L., Lu, D., Luo, J.-J., Qin, L., and Shen, Y.-Y. (2011). Abscisic acid plays an important role in the regulation of strawberry fruit ripening. Plant Physiology **157**: 188–199.

Krizek, B.A., Bequette, C.J., Xu, K., Blakley, I.C., Fu, Z.Q., Stratmann, J.W., and Loraine, A.E. (2016). RNA-seq Links the Transcription Factors AINTEGUMENTA and AINTEGUMENTA-LIKE6 to Cell Wall Remodeling and Plant Defense Pathways. Plant Physiology **171**: 2069–2084.

Kürsteiner, O., Dupuis, I., and Kuhlemeier, C. (2003). The *pyruvate decarboxylase1* gene of Arabidopsis is required during anoxia but not other environmental stresses. Plant Physiology **132**: 968–978.

Luo, J., Peng, F., Zhang, S., Xiao, Y., and Zhang, Y. (2020). The protein kinase FaSnRK1α regulates sucrose accumulation in strawberry fruits. Plant Physiol. Biochem. **151**: 369–377.

Merchante, C., Vallarino, J.G., Osorio, S., Aragüez, I., Villarreal, N., Ariza, M.T., Martínez, G.A., Medina-Escobar, N., Civello, M.P., Fernie, A.R., Botella, M.A., and Valpuesta, V. (2013). Ethylene is involved in strawberry fruit ripening in an organ-specific manner. J. Exp. Bot. **64**: 4421–4439.

Moyano, E., Martínez-Rivas, F.J., Blanco-Portales, R., Molina-Hidalgo, F.J., Ric-Varas, P., Matas-Arroyo, A.J., Caballero, J.L., Muñoz-Blanco, J., and Rodríguez-Franco, A. (2018). Genome-wide analysis of the NAC transcription factor family and their expression during the development and ripening of the Fragaria × ananassa fruits. PLoS ONE **13**: e0196953.

Mustroph, A., Barding, G.A., Kaiser, K.A., Larive, C.K., and Bailey-Serres, J. (2014). Characterization of distinct root and shoot responses to low-oxygen stress in Arabidopsis with a focus on primary C- and N-metabolism. Plant, Cell & Environment **83**: 2366–2380.

Sánchez-Sevilla, J.F., Vallarino, J.G., Osorio, S., Bombarely, A., Posé, D., Merchante, C., Botella, M.A., Amaya, I., and Valpuesta, V. (2017). Gene expression atlas of fruit ripening and transcriptome assembly from RNA-seq data in octoploid strawberry. Sci Rep **7**: 1–13.

Shen, Q., Chen, C.N., Brands, A., Pan, S.M., and Ho, T.H. (2001). The stress- and abscisic acid-induced barley gene HVA22: developmental regulation and homologues in diverse organisms. Plant Mol. Biol. **45**: 327–340.

Sugawara, S., Hishiyama, S., Jikumaru, Y., Hanada, A., Nishimura, T., Koshiba, T., Zhao, Y., Kamiya, Y., and Kasahara, H. (2009). Biochemical analyses of indole-3-acetaldoxime-dependent auxin biosynthesis in Arabidopsis. Proc. Natl. Acad. Sci. U.S.A. **106**: 5430–5435.

van der Linde, K., Gutsche, N., Leffers, H.-M., Lindermayr, C., Müller, B., Holtgrefe, S., and Scheibe, R. (2011). Regulation of plant cytosolic aldolase functions by redox-modifications. Plant Physiol. Biochem. **49**: 946–957.

Wang, H., Jones, B., Li, Z., Frasse, P., Delalande, C., Regad, F., Chaabouni, S., Latché, A., Pech, J.-C., and Bouzayen, M. (2005). The tomato Aux/IAA transcription factor IAA9 is involved in fruit development and leaf morphogenesis. Plant Cell **17**: 2676–2692.
