# Supplementary material for: The NAC transcription factor FaRIF controls fruit ripening in strawberry
Source: Plant Cell. 2021 Feb 24;33(5):1574–93. doi: 10.1093/plcell/koab070 (PMC8254488; doi:10.1093/plcell/koab070)
Supplement: koab070_Supplementary_Data [file koab070_supplementary_data.zip › tpc.00833.2020-s11.pdf]

The NAC Transcription Factor FaRIF Controls Fruit Ripening in Strawberry

Carmen Martín-Pizarro, José G. Vallarino, Sonia Osorio, Victoriano Meco, María Urrutia, Jeremy Pillet, Ana Casañal, Catharina Merchante, Iraida Amaya, Lothar Willmitzer, Alisdair R. Fernie, James J. Giovannoni, Miguel A. Botella, Victoriano Valpuesta, David Posé

Corresponding author: *David Posé* dpose@uma.es

Victoriano Valpuesta valpuesta@uma.es

Review timeline:

**TPC2020-RA-00833**	Submission received:	Oct. 06, 2020
	1st Decision:	Nov. 25, 2020 *request revisions*
**TPC2020-RA-00833R1**	1st Revision received:	Jan. 12, 2021
	2nd Decision:	Jan. 30, 2021 *accept pending minor revision*
	Final acceptance:	Feb. 20, 2021

REPORT: (The report shows the major requests for revision and author responses. Minor comments for revision and miscellaneous correspondence are not included. The original format may not be reflected in this compilation, but the reviewer comments and author responses are not edited, except to correct minor typographical or spelling errors that could be a source of ambiguity.)

TPC2020-RA-00833 Submission received Jan. 12, 2021

We have received reviews of your manuscript entitled "The NAC Transcription Factor FaRIF Controls Fruit Ripening in Strawberry." Thank you for submitting your best work to The Plant Cell. The editorial board agrees that the work you describe is substantive, falls within the scope of the journal, and may become acceptable for publication, pending revision and potential re-review.

The reviewers were quite supportive of this work and are enthusiastic about its contribution to our understanding of strawberry fruit ripening. In your revision, please pay attention to the use of particular transformants and rationale for why the individual lines were chosen for each assay. This was a concern raised by two of the reviewers and warrants some discussion. I would recommend language such as "# out of # individual transformant lines displayed X phenotype. Two representative lines are shown in Figure Y."

A note from the Editor-in-Chief: We are trying to make a concerted effort to change green/red comparisons to green/magenta to make our figures understandable to those with color vision deficiencies. We noticed that a small number of your figures utilize red/green contrasts, so as you prepare the final version of the figures, please check the figures for red/green color use. I recognize that you are trying to utilize colors in some of your graphs that match the colors of the fruit, so you may not need to change all of them. However, the following figures may need to be corrected and the red/green use isn't matched to the fruits: Figures 2A/C, 3A/B/E, 4A/B, 5A/B/C, and you might want to check over the Supplemental Figures for similar comparisons. Please note that color changes do not need to be highlighted or tracked in the revised manuscript, but could be noted in the cover letter or response document.

TPC2020-RA-00833R1 Revision received Jan. 12, 2021

-- Reviewer comments (with **Authors response in bold**):

Reviewer #1 (Comments for the Author):

The present work is very interesting for the advance in the knowledge of the ripening regulation of the fleshy fruits, and it is on the line of the description of master regulators of ripening. The authors have developed an extensive and deep analysis of the effects of the RIF gene on strawberry fruit ripening.

I have noted some points that could improve the presentation of the results and discussion:

Point 1.- If this NAC transcription factor controls strawberry fruit ripening, what about aroma regulation? Any data that support aroma regulation by FaRIF? I suggest including these data, if not, authors should discuss this point. I think including this could be better to support the role of FaRIF.

RESPONSE: We appreciate the suggestion. We did not include this information in the first submitted version because we did not validate the role of FaRIF in strawberry aroma through an analysis of volatiles. Nevertheless, we have analysed in our transgenic lines the expression of the main genes reported to be involved in the production of volatile compounds in strawberry. Our data show that FaRIF promotes the expression of genes involved in the biosynthesis of the phenylpropanoid volatile eugenol and the sesquiterpenes linalool and nerolidol. These data suggest that, although a volatilome analysis was not performed in this study, the general regulatory role of FaRIF in strawberry fruit ripening might include the control of fruit aroma.

Therefore, we have included the following changes:

-Line 76 (marked in blue): […] and the volatile compound eugenol (EMISSION OF BENZENOIDS II, EOBII, which acts together with the DOF-like TF FaDOF2) (Medina-Puche et al., 2015; Molina-Hidalgo et al., 2017).

-Line 225 (marked in blue): Besides these NAC genes, other ripening-related TF genes, as the R2R3 MYB TF *FaEOBII*, the DOF-like *FaDOF2*, and two bHLHs, i.e., *FaSPT* and *FaPRE1*, were also downregulated in the *35Spro:RIF-RNAi* lines (Supplemental Data Set 2).

-Line 256: The title of the section was modified to include this part (changes marked in blue): "FaRIF regulates the phenylpropanoid pathway, the accumulation of anthocyanins and lignin, and the expression of aroma-related genes"

-The following paragraph in line 295: "Volatiles play an important role in the aroma of strawberry fruits. The accumulation of these compounds is closely related to changes in secondary metabolism during fruit ripening. In order to identify a putative role of FaRIF in the regulation of volatile production, we analyzed the expression in receptacles of the *35Spro:RIF-RNAi* lines of a number of genes reported to be involved in volatile biosynthesis in strawberry. Interestingly, the expression of *FaEOBII* and the DOF-like TF *FaDOF2*, which act synergistically activating the transcription of the *EUGENOL SYNTHASE 2* (*FaEGS2*) to positively regulate the synthesis of the phenylpropanoid eugenol (Molina-Hidalgo et al., 2017), were downregulated in red fruits of the *35Spro:RIF-RNAi* lines (Supplemental Figure 8B). Furthermore, the expression of *FaEGS2* was also downregulated in the *FaRIF*-silenced ripe fruits (Supplemental Figure 8B). Moreover, the red receptacles of the *35Spro:RIF-RNAi* lines also showed a lower expression of the *NEROLIDOL SYNTHASE 1* (*FaNES1*) (Supplemental Figure 8B), involved in the synthesis of the terpenic volatile compounds linalool and nerolidol (Aharoni et al., 2004). These results suggest that FaRIF might be important to control the biosynthesis of compounds responsible for strawberry fruit aroma during the ripening process."

-Line 470 (marked in blue): […] and for genes involved in volatile production, hormone biosynthesis and signaling, and sugar and energy metabolism (Figure 6E). […]

Finally, in order to show these data, we have included an extra panel in the Supplemental Fig. 8 (8B), which displays the expression of the mentioned aroma-related genes in white and red receptacles of *35Spro:RIF-RNAi* and control lines. In addition, we have included a panel in Fig. 6E with the expression ratio of those genes in the *EXP2pro:RIF-RNAi* and *35Spro:RIF* lines.

Point 2.- Although *FaNAC035* expression is specific to fruit, I think the authors didn't comment about the phenotype of transformed plants in terms of possible effects in other non-fruit tissues.

RESPONSE: Indeed, the phenotype in non-fruit tissues was not included, and it is of interest to comment it in the publication. For this reason, a new figure (new Supplemental Figure 4) has been included. This figure shows the phenotype of adult plants of the transgenic and control lines studied in this work. In contrast to all the RNAi lines, which were indistinguishable from the control plant, both overexpression lines (*35Spro:RIF #1* and *35Spro:RIF-GFP #1*) were severely affected in its growth and development, suggesting that the ubiquitous expression of *FaRIF* has deleterious effects. We have included the following sentences in the text:

-Line 167: Constitutive silencing of *FaRIF* did not result in altered vegetative growth and development (Supplemental Figure 4).

-Line 429: In contrast to *35S*- and *EXP2*-RNAi lines, adult plants overexpressing *FaRIF* showed an impaired plant development (Supplemental Figure 4), suggesting that the ubiquitous expression of *FaRIF* has deleterious effects.

Point 3.- About cell wall-modifying genes, what would be the explanation for the upregulation of *PL2/plB, PG1, PME38,* and *RGL1* in RNAi lines? Some of this could be included in the Discussion section. The same for monolignol pathway-related genes (*HCT, CCR*).

RESPONSE: We do not have an easy explanation of the increased expression of these cell wall-modifying genes in the *FaRIF*-silenced receptacles, since they belong to gene families whose members are involved in different molecular processes, sometimes not directly related to cell wall disassembly. This is the case, for instance, of a cell wall related PME (*FaPE1/FaPME7*) which is involved in the production of demethylated oligogalacturonides rather than in the gross demethylation of pectins (Osorio, et al. Plant J, 2007, 54(1):43–55; Xue, et al. BMC Plant Biology, 2020, 20:13). Furthermore, within a gene family there are differences on the contribution of each enzyme to the total enzymatic activity. This is the case for instance of *FaPG1* and *FaPG2* (Quesada MA, et al. Plant Physiol, 2009, 150(2):1022–1032). A deeper knowledge of the activities and substrates of all these cell wall enzymes would be necessary to explain the effect of the up-regulation of them in RNAi lines.

Our work shows that fruit softening is promoted by FaRIF, since both *35S*- and *EXP2*-RNAi fruits were firmer, while in the overexpressing lines were softer. In parallel, we show that many genes reported to be involved in the cell wall disassembly showed an opposite expression pattern in the RNAi and overexpression lines, supporting a role of FaRIF in the promotion of cell wall disassembly. We believe that going beyond this point would be speculative, and would require a deep analysis of cell wall structure and composition. Nevertheless, to clarify this issue, we have modified the sentence in line 523 in Discussion as follows:

"Our transcriptomic analyses indicate that FaRIF regulates positively many genes involved in cell wall disassembling (Figures 3A and 6E), although some other cell wall-related genes

were upregulated in the RNAi lines (Supplemental Figure 8A). Therefore, although we cannot predict the specific effects of *FaRIF* silencing on the cell wall structure and composition, the altered firmness of the fruits in both the *FaRIF*-RNAi and overexpression lines supports a general role of FaRIF on the cell wall degradation and the loss of firmness during ripening."

Regarding the genes related to the monolignol pathway, we have clarified the effect that the changes on the expression of these genes might have in the pathway, adding the blue-marked sentence in line 273, and including also the reference to the *CAD9* gene, which was missing in the previous version.

"Conversely, genes of the monolignol pathway, such as those encoding for hydroxycinnamoyltransferase (*HCT*), the cinnamoyl-CoA reductase (*CCR*), and the cinnamyl alcohol dehydrogenase 9 (*CAD9*), were upregulated (Figure 3C), suggesting an enhanced activity of this branch of the pathway".

Point 4.- In Fig. 3C, is the $\log_2$ expression ratio of UFGT lost? I think data for this gene could be relevant for the RIF's control of anthocyanin accumulation. In this sense, if cyanidin hexose had a drastic reduction, why a* color parameter is apparently not affected in RNAi lines (Fig. 2C)?

RESPONSE: We appreciate the comment since we missed to show in Figure 3C the expression ratio for *UFGT*s. Our RNA-seq data revealed that several *UFGT*s were downregulated in the *35Spro:RIF*-RNAi red receptacles. Among them, the previously described FvH4_7g33840 (Lin-Wang et al., Frontiers in Plant Science, 2014,5(651)). However, this gene did not fulfill the statistic parameters used in this study, i.e. FDR≤0,05, to be considered differentially expressed (new Supplemental Data Set 2). Nevertheless, two other genes belonging to the *UFGT* family, FvH4_1g19440 and FvH4_7g15020 were significantly downregulated. Therefore, we have included in Fig. 3C the $\log_2$ of the ratio of the expression of RNAi/control for these two *UFGT* genes. We also included the expression of these *UFGTs*, named *FaUFGT1* and *FaUFGT2*, in the chart in Fig. 6E. Besides these two *UFGT* genes, we have also included in Fig. 3C the log2 expression ratio of a second *LAR* gene (FvH4_5g04260), which was also missing in the previous version. Finally, we have included in the Supplemental Table 2 the gene IDs for some phenylpropanoid-related genes (*CHI1*, *CHI2*, *F3H*, *DFR1*, *DFR2*, *F3'H*, *LARs*, *ANS*, *ANR1*, *ANR2*, *UFGT1*, *UFGT2*, *C3H* and *COMT*), information that was missing in the previous submitted version.

Regarding the lack of difference in the a* parameter between the control and *35S*-RNAi red receptacles, it is important to mention that, while pelargonidin is responsible of the orange-red color, cyanidin is responsible of red-magenta, being magenta in the midway between red and blue colors. The CIELAB a* parameter is relative to the green-red opponent colors (negative values toward green and positive values toward red), while the b* represents the blue-yellow opponents (negative numbers towards blue and positive toward yellow). Therefore, the similar levels of pelargonidin-glucose in control and *35S*-RNAi ripe receptacles might explain the similar a* values in those lines. However, the lower amount of cyanidin-glucoside in the *35S*-RNAi ripe receptacles (and therefore, with a reduction in the magenta and blue color) is consistent with the higher b* value (depleted in blue) observed.

Point 5.- Regarding ABA biosynthesis, what about the effect on the expression of the other two *NCED* genes? Is *FaNCED1* or *FaNCED2* expression affected?

RESPONSE: Three *NCEDs* have been shown to be expressed in strawberry fruits (Sánchez-Sevilla et al., 2017, Sci Rep, 7: 13737): *NCED3* (FvH4_3g16730), *NCED5*, also known as *NCED2*

(FvH4_3g05440), and *NCED6* (FvH4_4g05900). *NCED6* expression is extremely low (below 1 RPKM) in our transcriptome data. That is why it was not included in Supplemental Data 1. On the contrary, *NCED5* is the most expressed *NCED* in strawberry fruits. This gene was also downregulated at the red stage, however it was not included in the previous version since it was not significantly downregulated in *35Spro:RIF*-RNAi #3 (FDR p-value= 0.08). Now, the expression of the three *NCEDs* have been included in an extra sheet of the excel file for the new Supplemental Data Set 4, and clarified in line 306 of the text as follows (blue font):

"Interestingly, the gene encoding the rate-limiting enzyme in ABA biosynthesis, 9-cisepoxycarotenoid dioxygenase (*FaNCED3*), was significantly downregulated in the *FaRIF*silenced fruits at both ripening stages (Figure 4). Furthermore, the most expressed *NCED* in strawberry receptacles, *FaNCED5* (Liao et al., 2018), also named *FaNCED2* (Gu et al., 2019), was also downregulated in red receptacles of the silenced lines, although it was only significant in line #11 (Supplemental Data Set 4)."

Minor points:

Point 6.- A comment about projections of the work: the minor SSC values observed in *FaRIF* downregulated (or knockout) lines could work against fruit quality. Maybe authors could add this advice for breeding purposes.

RESPONSE: We agree with the reviewer that the reduced SSC values observed in the *35Spro:RIF-RNAi* lines works against fruit quality. However, the *EXP2pro:RIF-RNAi* lines did not show any alteration in SSC, but they did show an increase in fruit firmness, probably due to the late effect of the RNAi hairpin driven by the *EXP2* promoter. Therefore, we have included a sentence in line 590 to highlight this issue:

"Although the constitutive silencing of *FaRIF* reduced the SSC in ripe fruits, a late modulation of its expression, such as occurs in the *EXP2*-RNAi lines, might benefit from both getting an improved fruit firmness without changes in the sugar content."

Point 7.- line 67: I suggest replacing or add the reference Concha et al., 2013 (Plant Physiol. Biochem. 70, 433) since it is a broader analysis for the jasmonate effect on strawberry ripening.

RESPONSE: We have replaced the reference in this line and included Concha et al., 2013 (Plant Physiol. Biochem. 70, 433).

Point 8.- line 164: add "A" after Figure 2.

RESPONSE: We have added "A" after Figure 2.

Point 9.- lines 174-175: Authors comment that ripe achenes in the *35S:RIF*-RNAi lines were "also lighter in color"...where can this be observed? Any figure?

RESPONSE: We appreciate this comment. We have now included a "zoom-in" picture in Fig. 1D to show the lighter color phenotype of *35Spro:RIF-RNAi* achenes at the ripe stage.

Point 10.- lines 212-214: please indicate where this can be observed in figure 3.

RESPONSE: We have added the references "Figure 3A; Supplemental Data Set 4".

Point 11.- line 363: add "A" after Figure 5.

RESPONSE: We have added "A" after Figure 5.

Point 12.- lines 495-499: add Liao et al. 2018.

RESPONSE: We have added the reference of Liao et al., 2018 (PNAS. 115:E11542-E11550) in this paragraph.

Reviewer #2 (Comments for the Author):

In the manuscript entitled "The NAC Transcription Factor FaRIF Controls Fruit Ripening in Strawberry" by Martín-Pizarro et al the authors present a large set of results focusing on a strawberry NAC, NAC035 or RIF, which affects fruit ripening. The authors have identified by transcriptome analysis a candidate gene, and shown using diverse methods that this NAC affects fruit ripening, and the expression of a number of softening and phenylpropanoid related genes.

The manuscript is well written and there are only a few small errors (see below). However, there are a couple more important issues.

Major issues:

Point 1. Number of replicate lines - only two are chosen (of the *35Spro:RIF*-RNAi) for close analysis. More were made. What was the rationale for only doing two lines? It should be made clear (eg. line 191 onwards) the number of fruit per replicate.

RESPONSE: As a first step, we selected four *35Spro:RIF*-RNAi independent lines which displayed the same fruit phenotype (lighter red color and delayed ripening). However, only two representative lines were selected for all the comprehensive analysis (RNA-seq, metabolome studies, etc) based in their low *FaRIF* mRNA levels measured by qPCR.

To clarify this issue, we have included a picture of the ripe fruits of the control and the four independent lines (new Supplemental Figure 3), and the following sentence in line 162: "Four independent lines were transferred to the greenhouse and displayed a similar fruit phenotype (Supplemental Figure 3). Two of them were selected, i.e. *35Spro:RIF*-RNAi *#3* and *#11* as representative lines based on their low levels of *FaRIF* mRNA (Figure 2A)."

Regarding the number of fruits per replicate, some information was already included in the Material and Methods section and Figure legends. Nevertheless, we have included the missing information and clarify how many fruits per replicate was included in each experiment:

- **Figure 2 Legend: […] (F) Graphs representing the percentage of fruits in each developmental/ripening stage at each time point. Ten fruits were analyzed per genotype […].**
- **Figure 2 Legend: A mistake in the order of the number of replicates has been corrected in the legend of Figure 2. Now it reads: […] Data in (A) and (C) are means ±SE of three and ten biological replicates respectively analyzed by Student's t-test (*P<0,01; **P<0,001).**
- **Figure 6 Legend: […] (D) Representative pictures of a single fruit for control and *FaRIF* overexpression lines out of ten analyzed showing the progress of coloring during 14 days. […]**
- **Line 656 (M&M): […] Ten fruits from each line (control, *35Spro:RIF*-RNAi, *35Spro:RIF*, and *35Spro:RIF-GFP*) […].**

- **Line 666 (M&M): […] Each biological replicate was constituted by five receptacles. […]**
- **Line 682 (M&M): […] Each biological replicate was constituted by a minimum of 20 receptacles. […]**
- **Line 715 (M&M): […] Five replicates per condition and genotype were conducted.**

Point 2. Line 422 - only one line of *35Spro:RIF* and *35Spro:RIF-GFP* was analysed. Is this a representative line??

RESPONSE: We appreciate this question. Only one of each *FaRIF* overexpression line survived in the greenhouse. The two lines displayed a clear impaired overall development, indicating a deleterious effect when *FaRIF* is overexpressed. However, despite these defects, all the phenotypical analyses carried out in the fruits showed that these two overexpressing lines, although they differed in the presence of a GFP tag, were affected in the same way.

To clarify this issue, we included a new Supplemental Figure showing the adult plant phenotype (new Supplemental Figure 4), and the following changes in the main text (lines 422429):

"Two out of four established stable transgenic lines for *EXP2pro:RIF*-RNAi (#1 and #7) and the only two surviving overexpressing lines (*35Spro:RIF #1* and *35Spro:RIF-GFP #1*) were selected for further characterization (Figure 6). […] In contrast to *35S*- and *EXP2*-RNAi lines, adult plants overexpressing *FaRIF* showed an impaired plant development (Supplemental Figure 4), suggesting that the ubiquitous expression of *FaRIF* has deleterious effects."

Point 3. Genomics - line 200 - RNAseq was *Fragaria ananassa* laid down on *F. vesca* gene models. This should be made clearer. How much information was lost in this step? How is this corrected for?

RESPONSE: As the reviewer indicates, our transcriptome data were analysed by mapping the reads on the *F. vesca* genome. This was performed before *F.* x *ananassa*'s was published last year (Edger et al., 2019, Nat Genet, 51:541-547). Although *F. vesca* contributes with one of the subgenomes of *F.* x *ananassa*, it is true that using the octoploid genome to map the transcriptome data would help us to identify the expression of the homeologous genes in *F.* x *ananassa*. However, we discarded this possibility of re-analyzing the data for the following reasons:

1) **The *F.* x ananassa genome published by Edger et al. (2019) is the first and fully sequenced genome published to date. Although of excellent quality, especially in terms of assembly, it still requires annotation improvements. On the contrary, the annotation of the *F. vesca* genome is more complete than that of *F.* x *ananassa*. This is essential in transcriptome analyses to avoid misannotations, and therefore, inaccurate results and conclusions.**
2) **There is a high level of identity between orthologous genes in these two species, being at least over 93% (Bombarely et al., 2010; BMC Genomics, 11:503). Furthermore, both genomes have a high level of synteny (Edger et al., 2019; Hardigan et al., 2020, Frontiers in Plant Science, doi: 10.3389/fpls.2019.01789), facilitating the use of the diploid genome as the reference for the octoploid species**
3) **Edger et al. (2019) identified a dominant subgenome in *F.* x *ananassa*, which importantly is contributed by the *F. vesca* progenitor. Thus, the dominant *F. vesca* subgenome is more highly expressed than the other subgenomes, contributing to around two thirds of the entire transcriptome. This is also of great importance supporting the use of the diploid genome reference for the analysis of transcriptome data from *F.* x *ananassa.***
4) **The *F. vesca* genome has been used in transcriptome analyses performed previous to Edger's work, but, importantly, it is still being used and accepted in recent publications due to the previously mentioned reasons. Some examples are the following:**

Wang et al., (2020), Genomics. 112:2369-2378.

Luo et al., (2020), Mol Genet Genomics. 295:421-438

Medina-Puche et al., (2019). BMC Plant Biol. 19:586

Vallarino et al., (2019). Plant Biotechn J. 18:929-943

We believe that the use of the *F.* x *ananassa* genome as the reference for transcriptome studies in this species will be the option in a near future but, at present, we believe that using *F. vesca* genome is still a better option.

We have included in line 692 the sentence marked in blue in order to clarify the quality of the *F. vesca* genome: "Raw sequences were trimmed and mapped against using the assembly and annotation version v4.0.a1 of the *F. vesca* reference genome (https://www.rosacea.org/species/fragaria_vesca/genome_v4.0.a1; Edger et al., 2018), a high quality reference genome, using CLC Genomics Workbench 9 […]."

Point 4. Line 218 - NAC042 - is affected. This is one of the ripening induced NACs - is it on the phylogeny? If so, it should be clearer. Do the authors think this gene is affecting the phenotype - rather than NAC035?

RESPONSE: The *F. vesca* NAC042 is included in the phylogenetic tree. The tree was generated using the sequences of the cloned *FaNAC035* from *F.* x *ananassa,* and all the *F. vesca* NAC proteins described by Moyano et al. (2018). In the text we wrote the following (line 141): "A phylogenetic analysis of FaNAC035 using the 112 NAC proteins from *F. vesca* (Moyano et al., 2018) and additional NACs belonging to different subgroups based on their C-terminal domain was performed (Supplemental Figure 1).".

Regarding the second question, the general role in ripening regulation that plays FaRIF is the consequence of its function controlling the expression of direct targets, what includes regulatory genes, such as those encoding for other transcription factors. Our transcriptome data supported that FaRIF acts upstream of *NAC042* promoting its expression, since *NAC042* was significantly downregulated in both *35S$_{pro}$:RIF*-RNAi and *EXP2$_{pro}$:RIF*-RNAi lines, and upregulated in the overexpression lines. However, whether *NAC042* is a direct or an indirect target has not been experimentally validated in this work. Nevertheless, although we cannot discard that some of the transcriptome changes found in the *RIF*-RNAi and overexpression lines might be due to the altered expression of *NAC042*, it might be difficult to hypothesize that all the general defects in ripening that displayed the transgenic lines are the consequence of the changes in the *NAC042* expression. A functional analysis of this gene should be necessary to study the role of this TF and the extend of the processes *NAC042* regulates. Finally, it is also important to mention that NAC genes are able to interact as heterodimers to regulate the expression of their target genes, such as occurs for example in the case of *BLOOD* (*BL*) and *PpNAC1* in peach (Zhou, et al.; 2015, Plant J, 82, 105–121). Thus, it would be also possible that *NAC035* and *NAC042* might interact between each other to regulate strawberry fruit ripening. This possibility will be of great interest in future works.

Point 5. Line 258 onwards - anthocyanins show a large change. Is FaMYB10 affected?? It doesn't appear to be on the list of DEGs. Therefore, how is NAC035 acting on this pathway, if MYB10 is not a target?

RESPONSE: The expression of *FaMYB10* was not altered in ripe receptacles of the transgenic lines. Thus, our best explanation with our current data is that the some of the structural genes of the flavonoid pathway might be regulated by both FaMYB10 and FaRIF either independently

or in a coordinated way, since it has been reported the interaction between MYB- and NAC-like TFs (Trigg et al., 2017, Nature Methods, 14:819–825). Therefore, in these two situations, when the level of FaRIF protein is reduced, the transcription of the phenylpropanoid-related genes would be jeopardized. In order to shed light into this issue, it would be necessary to identify the target genes of FaRIF and FaMYB10 (ChIP-seq or DAP-seq for example) and to study the putative protein-protein interaction between these two TFs.

Point 6. Lin-Wang et al (2014; Engineering the anthocyanin regulatory complex of strawberry, Frontiers in Plant Science) reports the effects of knocking down MYB10. Are these affects seen in NAC035 knock-down lines??

RESPONSE: **Lin-Wang et al. performed a transcriptome analysis using mature fruits of wild type, and *35S:MYB10* and *35S:MYB10*-RNAi transgenic *F. vesca* plants. Comparing the differentially expressed genes in their dataset with ours, we have found that genes involved in the phenylpropanoid pathway, such as *PAL1*, *CHS*, *CHI*, *F3H*, *DFR2*, and *ANS*, were downregulated in both *35Spro:RIF*- and *35S$_{pro}$:MYB10*-RNAi ripe fruits. As previously mentioned, these data suggest that, since *MYB10* expression is not affected in ripe *35Spro:RIF*-RNAi receptacles, FaRIF might control the expression of these genes either independently of MYB10 or forming a complex with this TF. This would require future experiments as we have explained before.**

Furthermore, Lin-Wang et al. found that the amount of cyanidin, pelargonidin and coumaric acid hexose were significantly affected by the overexpression (increased levels) and knockdown (reduced levels) of *MYB10.* Our metabolomic data show that knocking down *FaRIF* also decreases the levels of cyanidin-hexose, but not that of pelargonidin-hexose, while it altered in the opposite way the content of coumaric acid hexose. Furthermore, mutations in *FvMYB10* reduced the level of ellagic acid hexose (Castillejo et al., 2020, Plant Cell, 32:3723-3749), while ellagic acid is also associated with MYB10 (Härtl, et al., 2017, Sci Rep. 7:45113; Wang et al., 2020, 18:1169-1184), as occurs in the *RIF*-RNAi lines. Therefore, we can hypothesize that, although these two TFs control the expression of some structural genes of the ellagic/phenylpropanoid pathway in the same way, they might also have different target genes and/or control differently some genes of these pathways.

Point 7. Figure 6 - the *35Spro:RIF* line (single line!) shows a dramatic dark phenotype. What anthocyanins are these?

RESPONSE: **As previously mentioned, the main anthocyanins in strawberry are pelargonidin- and cyanidinglucoside. The significant reduction in the levels of cyanidin-hexose in the *35Spro:RIF-RNAi* ripe receptacles led us to hypothesize that the dark color in the overexpression lines might be due to an increase in the levels of this anthocyanin. Furthermore, the overexpression lines also showed an increase in the b* parameter of the CIELAB color space, supporting higher levels of the cyanidin-glucoside. However, since we did not perform a metabolomic analysis to validate it experimentally, we did not include this hypothesis in the main text.**

Minor issues:

Point 8. Abstract - Line 43 - should be "sugar accumulation", singular

RESPONSE: **The typo has been corrected.**

Point 9. Introduction - line 68 - yes, strawberries are non-climacteric, but do they "respond" to exogenous ethylene? Some statement on this should be made.

RESPONSE: We have added this sentence in line 60: "Nevertheless, some reports have found that the application of ethylene to strawberry fruits and the generation of plants with reduced ethylene sensitivity had an effect on strawberry ripening (Trainotti et al., 2005; Villareal et al., 2010; Sun et al., 2013; Merchante et al., 2013)."

Point 10. line 74 - date missing in Medina-Puche reference.

RESPONSE: This typo has been corrected. We have included the date (2013). We also corrected other dates missing in de Oliveira et al., 2011 (line 92), and Csukasi et al., 2011 (line 496) references.

Point 11. line 94 - mention of Nieuwenhuizen et al (2015) NACs in kiwifruit (Plant Physiology)

RESPONSE: The reference has been included in this line.

Point 12. Line 102 - FcNAC1 - is this in the author phylogeny?

RESPONSE: The protein sequence of FcNAC1 was not originally included in the phylogenetic analysis since it was included its ortholog in *F. vesca*: FvNAC022 (FvH4_3g08490). FvNAC022 and FcNAC1 differ only in 2 amino acids. This is mentioned in the text (lines 101 and 481). In line 101, we have clarified that *FcNAC1* is the ortholog to both *F. vesca* and *ananassa*'s *NAC022*.

Point 13. Results - line 132 - these ripening induced *NAC*s - are any the equivalent of the published *FcNAC1*? (the *ananassa* version of this)?

RESPONSE: Moyano et al (2018) named *FaNAC022* to the closest homolog to *F. vesca*'s *FvNAC022* in *F.* x *ananassa*. Therefore, *FcNAC1* is the closest gene to both *FvNAC022* and *FaNAC022*. To clarify this issue, we have included the blue part and removed the crossed-out part of the following sentences:

-Line 100: "Among them, the expression of *FcNAC1*, the ortholog of *F. vesca* and *ananassa*'s *NAC022* in *F. chiloensis*, ~~one of the parental species of the commercial strawberry *F. x ananassa*,~~ responded to ABA and auxin"

-Line 481: In this sentence, we have changed *FveNAC022* by *NAC022* in order to be more general (NAC022 refers to both *F. vesca* and *F.* x *ananassa*): "Among these ripening-related genes, only *FcNAC1*, the ortholog to *NAC022* in *F. chiloensis*, has been characterized in relation to strawberry ripening."

Point 14.- line 133 - For the general reader - a sentence describing what the receptical and achene is - this is a very strawberry-specific term.

RESPONSE: We agree with this suggestion. We have including the following sentence in line 54: "The strawberry fruit is an achenetum, consisting of a fleshy part (receptacle) that results from the development of the flower receptacle, in which the actual fruits (achenes) are embedded (Liu et al., 2020)."

Point 15. Line 351 - Carbohydrate metabolism - not "Carbohydrates".

RESPONSE: This typo has been corrected.

Point 16. Line 665 and 671 - "De-achened" - is a very strawberry-centric word.

RESPONSE: "Deachened receptacles" was substituted by "receptacles after the removal of achenes in line 665 and 671.

Reviewer #3 (Comments for the Author):

This manuscript describes the functional validation of a novel strawberry ripening NAC transcription factor regulator, FaRIF. The authors use a classical approach with both RNAi and overexpression in the host plant to determine the extent of its regulation. The use various appropriate techniques and data help to strengthen the hypothesis. The phenotypic analysis confirms this gene as a master regulator, having effects on both primary and secondary metabolism. The authors show how this is likely partly mediated by the effect on ABA, the major hormonal signal for strawberry ripening, and quite how this is actually achieved will be interesting for future study. Similarly, the direct effect that FaRIF has on other transcription factors in the regulatory cascade of fruit ripening will be of interest.

Point 1. I think a more detailed developmental series would perhaps have been more illuminating - the use of just two data points is not ideal, although the results do fit with the visual and chemical phenotype.

RESPONSE: We agree with the reviewer that performing a time-course experiment using more developmental/ripening stages would have been more informative, especially to know the role of FaRIF at early stages, such as the green stage. However, in this work we decided to focus in two of the ripening stages (white and red) since they represent well established developmental stages in relation to the ripening of the fruit. Furthermore, the expression of *FaRIF* is higher at these two stages, so it is expected to get a broader phenotypical and molecular alteration in the silenced lines compared to the control.

Point 2. While the use of two independent transgenic lines is of course acceptable, it would have been useful to see results from other lines in supplemental data to gauge the effect of FaRIF transcript level on phenotype.

RESPONSE: We appreciate this comment. It is a point that it was also raised by Reviewer #2. To clarify this issue, we have included a picture of the ripe fruits of the control and four independent *$35S_{pro}$:RIF*-RNAi lines (new Supplemental Figure 3), and the following sentence in line 162: "Four independent lines were transferred to the greenhouse and displayed a similar fruit phenotype (Supplemental Figure 3). Two of them were selected, i.e. *$35S_{pro}$:RIF-RNAi #3* and *#11* as representative lines based on their low levels of *FaRIF* mRNA (Figure 2A)."

Furthermore, we have included in line 422 the total number of *EXP2pro:RIF*-RNAi established: "Two out of four established stable transgenic lines for *EXP2pro:RIF*-RNAi (#1 and #7) […]"

Finally, we have also included a new supplemental figure displaying the adult plant phenotype (new Supplemental Figure 4) and clarified in the text why only one single line per each FaRIFoverexpression strategy was selected:

-Line 423: "[…] and the only two surviving overexpressing lines (*35Spro:RIF #1* and *35Spro:RIF-GFP #1*) were selected for further characterization (Figure 6)."

-Line 429: "In contrast to *35S-* and *EXP2*-RNAi lines, adult plants overexpressing *FaRIF* showed an impaired plant development (Supplemental Figure 4), suggesting that the ubiquitous expression of *FaRIF* has deleterious effects."

Point 3. I'm not sure why chosen to use the non-specific hexose when referring to cyanidin or pelargonidin? With LC-MS it is easy to distinguish which glycoside is present.

RESPONSE: The identification of secondary metabolites was performed using published data that do not specify the hexose. Therefore, although it is described that glucose is the main sugar bound to the anthocyanidins pelargonidin and cyanidin in strawberry, resulting in the main anthocyanins pelargonidin- and cyanidin-glucoside, we cannot identify which particular hexose is attached to the respective anthocyanidin. To clarify this issue, we have now included more detailed information about how the metabolite annotation was performed for both, primary and secondary metabolic profiling (line 699): "[…] For primary metabolites, the metabolite identification was based on cross-referenced with the Golm Data Base (Kopka et al., 2005). For secondary metabolites, putative identification and annotation were performed using literature, mainly from strawberry. Here, the data were presented as peak response in mass chromatograms and represented as a direct amount of peak area. No quantitative standards were used in this study. […]"

Point 4. The comment about the possible direct regulation of NAC42 is interesting (Line 222-225). In that the authors state that the sequence is 'rather different', it would help if this was proven experimentally.

RESPONSE: We agree with the comment of the reviewer. But, the regulation of *NAC042* expression by FaRIF is just a hypothesis that we have not validated. Therefore, we did not assume in the text that it is a direct regulation. Accordingly, we wrote (line 22): "[…] the hairpin sequence is rather different than the sequences of *FaNAC042*, suggesting that *FaNAC042* expression is regulated either directly or indirectly by FaRIF instead of an unspecific silencing."

And in line 461: […] The opposite expression of *FaNAC042* in the RNAi and the overexpression line supports the role of FaRIF promoting *FaNAC042* expression. […]

Our hypothesis is based on the following: if the downregulation of *FaNAC042* in the RNAi lines would have been due to an unspecific silencing of the *FaRIF*-RNAi hairpin, it would not be expected an upregulation of *FaNAC042* in the *35Spro:RIF* line, as it actually occurred. That is why we hypothesize that *FaNAC042* is a direct or an indirect target of FaRIF. The identification of direct targets of FaRIF is of utmost interest for us and will be studied in the future.

Point 5. I assume that FaRIF is homologous to apple NAC18? There are certainly sequence similarities and given the number of recent publications around this other Rosaceous gene I'm surprised that is has not been mentioned or discussed. Some of the English requires improvement but otherwise the manuscript is enjoyable to read, well prepared and the data is convincingly presented.

RESPONSE: We appreciate this suggestion. FaRIF and NAC18.1 (MD03G1222600) protein sequences have an identity of 63,38%. We have therefore included a reference for this TF in line 94: Members of this family have been reported to be involved in the regulation of ripening-associated processes in fruits such as […] apple (Yeats et al., 2019; Zhang et al., 2020).

Another reference to this TF have been included (marked in blue) in the Discussion section (line 575): "Furthermore, the phylogenetic analysis also showed homology between FaRIF and SlNOR, TF previously reported controlling tomato fruit ripening (Giovannoni, 2004; Wang

et al., 2019). Besides, it has been reported that NAC18.1, the ortholog to SlNOR in apple, regulates apple fruit ripening controlling fruit firmness and harvest time (Migicovsky et al., 2016; Yeats et al., 2019). Thus, all these data support a role of these NAC TFs in the regulation of ripening not only in both dry and fleshy fruits, but also in organs with different ontogenetic origins such as the tomato fruit, and false fruits such as apple (pome) and strawberry (achenetum).”

TPC2020-RA-00833R1 2nd Editorial Decision – *accept* **Jan. 30, 2021**

We are pleased to inform you that your paper entitled "The NAC Transcription Factor FaRIF Controls Fruit Ripening in Strawberry" has been accepted for publication in The Plant Cell, pending a final minor editorial review by journal staff. At this stage, your manuscript will be evaluated by a Science Editor with respect to its presentation of scientific content, compliance with journal policies, and presentation for a broad readership - you will be able to address the minor comment by Reviewer #1 at this stage.

Final acceptance from Science Editor **Feb. 20, 2021**
